# Engineering Intramolecular Compactness in Water-Soluble Single-Chain Nanoparticles for Optimized Platinum Delivery

**DOI:** 10.1021/acspolymersau.6c00083

**Published:** 2026-06-24

**Authors:** Miriam B. Lossa, Thorsten Hofe, Martina H. Stenzel

**Affiliations:** † School of Chemistry, 7800 University of New South Wales, Sydney 2052, NSW, Australia; ‡ 59971 Agilent Technologies Deutschland GmbH, Mainz 51120, Germany

**Keywords:** single-chain nanoparticles, platinum drugs, SAXS, intrinsic viscosity, compactness, intramolecular folding, ultrasmall polymer nanoparticles, SEC, drug delivery

## Abstract

Single-chain nanoparticles (SCNPs) have been extensively studied for various applications, including catalysis, drug delivery, antimicrobial agents, rheology modifiers, and in nanocomposites. For catalytic applications, the collapsed polymer chain was often cross-linked using a metal complex, which also served as the active catalytic site. Inspired by the successful formation of a metal complex containing SCNPs, we have adopted this system for the delivery of the anticancer drug cisplatin. However, to deliver cisplatin into cells across the cell membrane, an understanding of the detailed structure and compactness of SCNPs is essential. In this work, we synthesized a series of statistical copolymers based on polyethylene glycol methyl ether acrylate (PEGMEA) as water-soluble monomer, 2-carboxy ethyl acrylate (CEA) as the ligand for platinum drug conjugation, and a hydrophobic monomer (methyl acrylate (MA), butyl acrylate (BA), and benzyl acrylate (BzA)) to enhance the compactness of the SCNP. The library of polymers was conjugated with cisplatin, and the resulting SCNPs were characterized predominantly by size-exclusion chromatography (SEC) with viscosity detection and small-angle X-ray scattering (SAXS), as dynamic light scattering alone is insufficient to provide information about the SCNP formation. Increasing the amount of CEA and, therefore, the amount of cisplatin reduced viscosity and the radius of gyration (*R*_g_), thus increasing compaction. The same was achieved by incorporating hydrophobic comonomers with BA polymers showing the strongest collapse. The toxicity of these drug-loaded SCNPs was tested in the ovarian cancer cell line A2780, healthy lung cells MRC-5, and cisplatin-resistant ovarian cancer cells A2780cis, showing that greater SCNP collapse correlates with higher toxicity, but only in the absence of hydrophobic comonomers.

## Graphical abstract

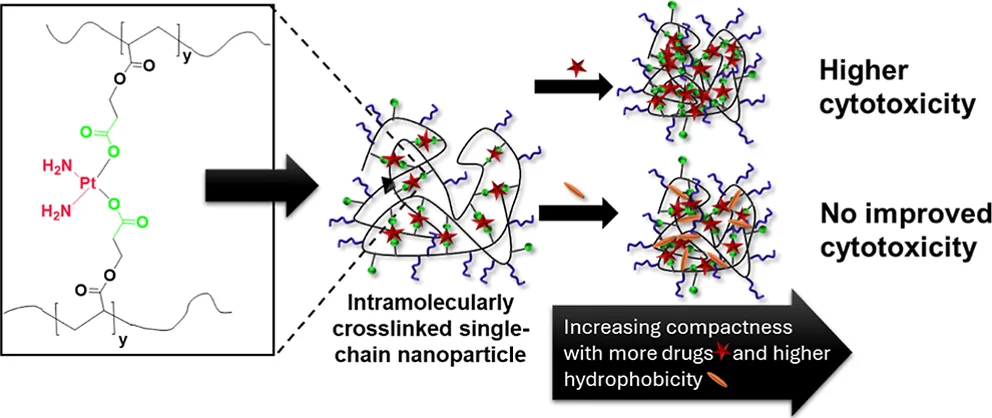

## Introduction

Ultrasmall nanoparticles for drug delivery appear to have a range of advantages, such as reduced protein corona formation,^1^ better penetration into the tumors,^2,3^ and enhanced circulation time in the bloodstream.^4,5^ However, very small NPs below 5 nm are rapidly removed by renal filtration.^6,7^ In this sweet spot of 5–10 nm, we can find single-chain polymer nanoparticles (SCNPs) that have drawn attention in the past decade as the next generation of drug delivery systems for combating cancer.^8,9^ The tunability of their supramolecular design enabled SCNPs to advance as a promising therapeutic approach for targeted drug delivery.^10–14^ An advantage of SCNP is their ability to deliver drugs directly into the cytosol, potentially bypassing drug-resistant mechanisms.^13,15,16^ Initial studies on SCNPs indicate that they can traverse cell membranes, a process influenced by their size, chain mobility, and hydrophobic–hydrophilic ratio.^15–17^ Especially, the latter is an important point to consider, as SCNPs destined for drug delivery will carry hydrophobic drugs that will change the properties of the SCNPs.^18^ The amount of work describing SCNP loaded with drugs is limited,^18–20^ most likely due to the complexity between drug loading and the immediate effect on the SCNP properties that will affect drug delivery.

Although the literature is rich with SCNPs that were obtained by metal complexation,^21–26^ only a few metal-based SCNPs have been explored for medical applications, such as in the case of Cu- ^26,27^ and Pd-containing polymers.^28^ This is surprising considering that metal-based drugs are widely used in clinical practice for the diagnosis and treatment of a broad range of diseases.^29^ However, so far, most metal-containing nanoparticles described in the literature are larger than 50 nm. A landmark study has shown that smaller nanoparticles used to deliver platinum drugs are superior at penetrating tumor tissue.^30^ It is therefore conceivable that platinum drug-containing SCNPs could be superior to existing nanoparticles that contain platinum drugs.

Before designing SCNPs, we need to identify key parameters, such as the degree of compaction and the relationship between the structure and morphology, to fully elucidate cell uptake and biological activity.^15^ It was shown that efficient cellular uptake can be achieved by suitable SCNP compaction^15^ and the right balance between hydrophobicity and hydrophilicity.^16,18^ In short, we need to understand the chemical composition and the structure to help optimize the biological activity. This requires thorough characterizations of SCNPs. Over the years, an effective toolset has been developed to characterize the physicochemical properties of sub-10 nm particles and to determine their compactness.^31–34^ Commonly employed characterization techniques to assess reduction in size upon intrachain collapse are dynamic light scattering (DLS),^35,36^ multiangle light scattering (MALS),^37–41^ DOSY-NMR,^36,42,43^ size exclusion chromatography (SEC),^34–37^ small-angle scattering (SAS),^44–47^ and asymmetrical field flow fractionation (AF4).^48^ Less widely applied is the measurement of the intrinsic viscosity of nanoparticles in solution,^32,49^ although some research groups use this technology to confirm the compaction of SCNPs routinely.^38–41,50–52^ A major advantage of online viscometry, also referred to as process or inline viscometry, is that it provides absolute data on the differential pressure between the pure solvent and polymer solution in a capillary system, which is further related to the intrinsic viscosity of the sample and therefore expresses the density of the macromolecule, given that the sample is dissolved in a theta solvent. Therefore, to accurately determine narrowly distributed polymer precursors and thus the degree of compaction, size, and shape of the resulting SCNPs with common lab benchtop solutions is limited by the lack of sensitivity or unsuitability within the ultrasmall size range (i.e., sub 10 nm).^32,48,49^

In this work, we aim to explore how we can control the compactness of SCNP and how this may affect the toxicity of the attached platinum drugs. We first designed a linear statistical copolymer system via RAFT polymerization composed of polyethylene glycol methyl ether acrylate (PEGMEA), which is widely used in drug delivery, and 2-carboxyethyl acrylate (CEA), which will serve as the ligand for the platinum drug (Scheme 1).^53^ The amount of CEA groups will not only determine how many potential cross-linking sites are available but also how much hydrophobic drug can be conjugated. Here, a range of processes will take place simultaneously: Carboxylate groups are essential for platinum drug conjugation, but the presence of negatively charged functionalities will also prevent high compaction. However, every attached platinum drug, which is considered hydrophobic, will render the polymer more hydrophobic and therefore counteract the repulsive forces of the carboxylates. To further enhance compactness, hydrophobic comonomers were incorporated. In 1995, the folding behavior of amphiphilic statistical polymer chains was first investigated by Morishima and co-workers,^54^ who showed that the introduction of hydrophobic groups can promote precollapse of individual polymer chains in an aqueous environment.^12^ Since then, a range of amphiphilic polymers that self-fold in structures reminiscent of core–shell particles have been reported.^55–58^ Therefore, methyl acrylate (MA), butyl acrylate (BA), and benzyl acrylate (BzA), which can potentially undergo π–π stacking, were employed as third comonomers at different molar ratios to the overall degree of polymerization (DP) (i.e., 10–40 mol %). The toxicity of the resulting platinum drug-containing SCNP will then be correlated with its compactness.

**Scheme 1:**
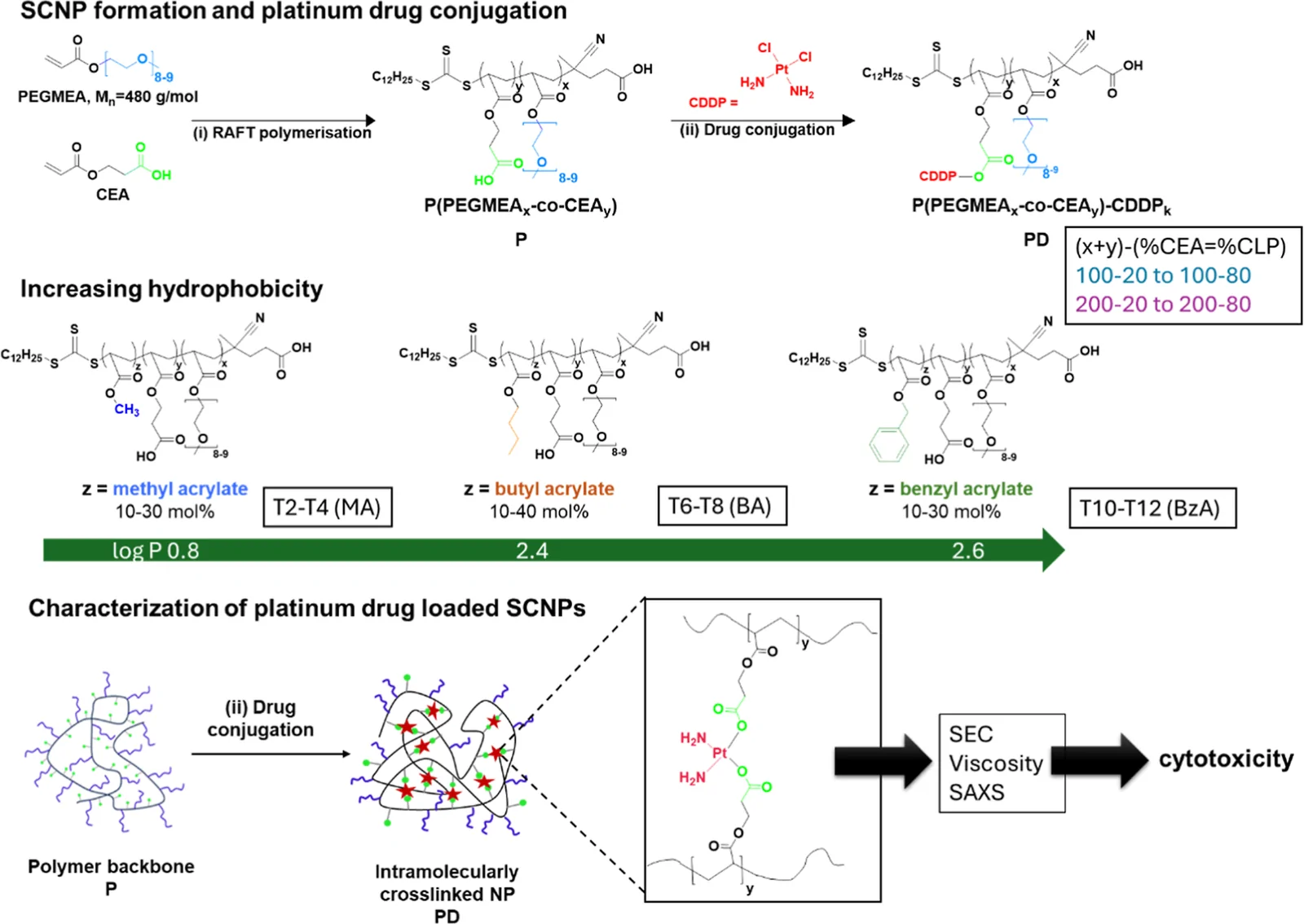
RAFT Synthesis and Cisplatin Conjugation of Polymer P ^a^

a Synthesis of polymer backbone P via RAFT polymerization (i) with different hydrophobicities (HM = hydrophobic monomer) and subsequent conjugation of cisplatin onto the polymer chain (ii), resulting in a polymer–drug conjugate PD bearing the structure of a single-chain nanoparticle upon intramolecular crosslinking of individual polymer chains or in multichain complexes, aggregates, or non-crosslinked particles. Scheme of polymer collapse upon crosslinking via metal–ligand complexation with cisplatin and the carboxylic acid groups on the polymer chain into highly compact single-chain nanoparticles (SCNPs). The label for each polymer is provided in Table 1.

## Experimental Section

### Materials

Unless otherwise specified, all chemicals were of reagent grade and were used as received: dimethylformamide (DMF; Chem Supply), diethyl ether (DEE; Chem Supply), disodium dihydrogen orthophosphate buffer (pH 8.7–9.3, Chem Supply), chloroform-d (CDCl_3_; Cambridge Isotope Laboratories, Inc.), and *N*,*N*′-dimethylacetamide (DMAc; Chem Supply). Lithium bromide (LiBr), butylated hydroxytoluene (BHT), 2-carboxyethyl acrylate (CEA), poly­(ethylene glycol) methyl ether acrylate (PEGMEA), methyl acrylate (MA), *n*-butyl acrylate (BA), *N*,*N*-dimethylformamide (DMF; peptide synthesis grade), phosphate-buffered saline (PBS), and *cis*-diamminedichloroplatinum (CDDP) were purchased from Sigma-Aldrich. The inhibitor contained in the monomer solutions was removed by running the liquid over the inhibitor removal (by Sigma-Aldrich) packed in a syringe before use. Azobis­(isobutyronitrile) (AIBN) was purchased from Fluka and purified by recrystallization from methanol twice. 4-Cyano-4-[(dodecylsulfanylthiocarbonyl)­sulfanyl] pentanoic acid (CDSTS) was purchased from Boron Molecular, benzyl acrylate (BzA) was purchased from AmBeed, and sodium hydroxide (NaOH), sodium azide (NaN_3_), and disodium dihydrogen orthophosphate anhydrous powder were purchased from Chem-Supply. HPLC-grade methanol (MeOH) was purchased from Fischer, HPLC-grade acetonitrile (MeCN) was purchased from RCI LabScan, and MQ Water was generated via a Milli Q IQ 7000 Ultrapure Lab Water System and a Biopak filter by Merck.

### Synthesis

#### RAFT Polymerization of P­(PEGMEA-*co*-CEA)

Statistical copolymers of polyethylene glycol methyl ether acrylate (PEGMEA) and 2-carboxyethyl acrylate (CEA) were prepared using 4-cyano-4-[(dodecylsulfanylthiocarbonyl)-sulfanyl]­pentanoic acid (CDSTS) as the chain-transfer agent (CTA). As an example, aiming for 40 mol % PEGMEA and 60 mol % CEA, the polymer was synthesized as follows: In a 10 mL round-bottom flask, 2 mL of a 2 M PEGMEA stock solution in DMF (4 mmol), 3 mL of a 2 M CEA stock solution in DMF (6 mmol), 0.2 mL of 0.25 M CDSTS stock solution (5·10^–2^ mmol), and 0.1 mL from 50 mM AIBN stock solution (5·10^–3^ mmol) were dissolved in a total volume of 5 mL DMF (grade for peptide synthesis). After purging the solution with dry N_2_, it was heated to 70 °C while stirring. After 8 h, the reaction was stopped by quickly immersing the vial into an ice bath and removing the septum, allowing oxygen to enter. The polymer was precipitated three times in cold diethyl ether and dried under vacuum at 40 °C. ^1^H NMR and SEC measurements were used to characterize the monomer conversion, ${\mathrm{M̅}}_{n}$ , and *D̵* of the obtained copolymers.

#### RAFT Polymerization of P­(PEGMEA-*co*-CEA-*co*-MA), P­(PEGMEA-*co*-CEA-*co*-BA), and P­(PEGMEA-*co*-CEA-*co*-BzAs)

Statistical copolymers of polyethylene glycol methyl ether acrylate (PEGMEA) and 2-carboxyethyl acrylate (CEA) with varying additional hydrophobic monomers (i.e., MA, BA, or BzA) were prepared using 4-cyano-4-[(dodecylsulfanylthiocarbonyl)-sulfanyl]­pentanoic acid (CDSTS) as the chain transfer agent (CTA). For example, aiming for 60 mol % PEGMEA, 30 mol % CEA, and 10 mol % MA, the polymer was synthesized as follows: In a 50 mL round-bottom flask, 3 mL of a 2 M PEGMEA stock solution in DMF (6 mmol), 2 mL of a 2 M CEA stock solution in DMF (4 mmol), 0.66 mL of a 2 M MA stock solution, 0.2 mL of 0.25 M CDSTS stock solution (5·10^–2^ mmol), and 0.1 mL from 50 mM AIBN stock solution (5·10^–3^ mmol) were dissolved in a total volume of 10 mL DMF (grade for peptide synthesis). The inhibitor in the monomer was removed by running the liquid over a column of basic alumina. Potential free amines in the DMF were removed by passing it through silica or by applying vacuum for a short time. After purging the solution with dry N_2_, the reaction mixture was stirred at 70 °C. After 8 h, the reaction was stopped by quickly immersing the flasks on ice and removing the septum, allowing oxygen to enter. The polymer was precipitated three times in cold diethyl ether and dried under vacuum at 40 °C. ^1^H NMR in CDCl_3_ and DMF SEC measurements were used to characterize the monomer conversion, ${\mathrm{M̅}}_{n}$ , and *D̵* of the obtained copolymers, respectively.

All polymers were similarly synthesized with variations in the volume pipetted from the 2 M monomer stock solution. Since every monomer stock solution was prepared at 2 M, the comonomer molar ratio was adjusted by varying the volume pipetted from the stock solution, aiming for a total comonomer concentration of 1 mol/L. For the copolymers consisting of three comonomers, the ratio between CEA and PEGMEA was kept at 50/50 of the remaining molar ratio to the third comonomer; e.g., 10 mol % methyl acrylate reaction solution consisted of 2 mL of 2 M CEA (4 mmol), 2 mL of 2 M PEGMEA (4 mmol), and 0.5 mL of 2 M MA (1 mmol).

### Polymer-Drug Conjugation with Cisplatin

Different amounts of cisplatin were conjugated to the polymer precursor. Conjugation of cisplatin was described in a previous work; however, a couple of modifications have been introduced here.^53^ Specifically, sample 200–50 (Table 1) was prepared by suspending 3.9 mg CDDP in 3.9 mL Milli-Q water (1.3·10^–2^ mmol; 1 mg·mL^–1^ CDDP) and sonicated for 30 min in the dark. The mixture was kept in the dark at room temperature overnight (*t* ≥ 12 h). 7.9 mg of 200–50 with carboxyl groups (1.3·10^–2^ mmol COOH) were dissolved in 0.6 mL of a 1 mg·mL^–1^ NaOH solution until dissolved. The polymer was added to the CDDP aqueous solution (3.9 mL), yielding a molar feed ratio of [1:1] COOH to CDDP. The mixture was immediately diluted to a final polymer-based concentration of 0.4 mg·mL^–1^ to prevent the formation of aggregates and interchain cross-linked particles. The mixture was left to react in a water bath at 37 °C for 8 h with gentle shaking. The product was purified by dialysis (MWCO 3.5 kDa) against Milli-Q water to remove excess amounts of free cisplatin.

**Table 1:** Summary of Statistical Copolymers Consisting of PEGMEA, CEA, and Hydrophobic Monomer Repeating Units (i.e., MA, BA, or BzA)^d^

polymer	RU PEG	RU CEA	RU HM	HM (%)	mol % COOH = CLP	${\overset{¯}{M}}_{n,theo.}$ (kDa)	${\mathrm{M̅}}_{n,\mathrm{SEC}}$ ^a^ (kDa)	*D̵* ^b^	molar ratio^c^ (CDDP/COOH)	DLE^c^ (%)	DLC^c^ (wt %)
100–20	71	18			20%	36.6	16.4	1.31	1:3.6	26.5	3.4
100–30	62	27			30%	33.6	19.2	1.32	1:2.2	44.6	8.8
100–40	52	36			41%	30.3	20.0	1.33	1:2.3	43.3	12.2
100–50	41	42			51%	25.6	21.7	1.27	1:2.4	41.2	15.3
100–60	35	54			61%	66.3	20.9	1.27	1:2.6	38.1	18.2
100–70	25	60			71%	20.5	22.3	1.28	1:3.4	28.7	18.6
100–80	16	69			81%	17.7	21.9	1.25	1:3.7	26.2	22.0
200–20	145	38			21%	75.3	22.8	1.40	1:3.1	32.9	4.2
200–30	122	54			31%	66.3	37.5	1.45	1:2.2	45.7	9.0
200–40	103	69			40%	59.4	31.2	1.52	1:2.3	44.5	12.0
200–50	85	88			51%	53.6	34.1	1.40	1:2.7	37.0	13.7
200–60	69	106			61%	48.5	37.9	1.35	1:2.7	37.3	17.8
200–70	52	123			70%	42.6	35.8	1.44	1:3.0	32.4	20.2
200–80	34	141			81%	36.6	39.4	1.31	1:3.8	27.2	21.4
T2 (MA)	70	77	17	10	47%	45.9	36.6	1.14	1:1.3	79.2	40.0
T3 (MA)	59	67	31	20	43%	40.5	34.9	1.14	1:1.3	80.6	39.9
T4 (MA)	49	58	48	31	37%	36.3	33.9	1.13	1:1.2	81.0	38.7
T6 (BA)	62	62	16	11	44%	40.5	36.2	1.13	1:1.3	75.8	39.0
T7 (BA)	66	67	36	21	40%	46.2	35.1	1.13	1:1.4	70.1	51.2
T8 (BA)	49	50	52	34	33%	37.4	32.6	1.12	1:1.2	82.0	32.8
T10 (BzA)	78	78	19	11	45%	51.9	41.2	1.15	1:1.6	62.8	57.9
T11 (BzA)	69	68	38	22	39%	49.1	38.4	1.14	1:1.2	82.4	34.3
T12 (BzA)	63	64	58	31	35%	48.9	33.2	1.12	1:1.2	81.3	32.2

a Determined from PEG/PEO calibration in 0.1 M phosphate buffer at pH 8.7–9.3 in water via SEC-RI.

b Determined from PMMA calibration in DMF with 2.5 g·L^–1^ LiBr and 0.1 g·L^–1^ BHT.

c Actual ratio measured using ICP-OES.

d The repeating units (RUs) are based on the conversion results from ^1^H NMR, the theoretical Mn was calculated using monomer conversions, ${\mathrm{M̅}}_{n,\mathrm{SEC}}$ , and dispersity (*D̵*) were determined through SEC in water and DMF, respectively. The DLE refers to the PD2 SCNPs obtained from a feed ratio of [CDDP]:[COOH] = 1:1, the PD1 SCNP results can be found in Supporting Information, Table S1.

### Drug Release of Cisplatin from SCNPs

The release of cisplatin from the Pt-SCNPs was determined using SEC with a UV detector set to 290 nm. All samples were prepared similarly to the following example: 750 μL of SCNP solution (0.4 mg·mL^–1^) was transferred into an Agilent autosampler vial (capacity of 2 mL). 750 μL of 2X PBS (pH 7.4) was added to the sample given in the vial, and the mixture (1:1 v/v) was gently shaken. To record the kinetics of the drug release, an aqueous SEC was employed using the Agilent 1220 Infinity Compact LC system comprising an LC pump (G4281B), an autosampler (G4282B), a column oven (G4283A), a multiple wavelength detector (MWD, G4284B), and a diode array detector (Infinity 1260 DAD WR, G7115A). The detector wavelengths were set to 290 nm to detect free cisplatin at its maximum absorbance and at 320 nm for the signal deriving from the trithiocarbonate RAFT agent on the polymer backbone. An 8 × 50 mm SUPREMA 5 μm guard column and an 8 × 300 mm SUPREMA linM 5 μm column were used for separation, while a 0.2 g·L^–1^ NaN_3_ aqueous solution was used as a mobile phase. The Agilent WinGPC was employed to control the LC system, command injections at selected time points for kinetic studies, and collect and evaluate data for the measured samples. The free platinum drug was observed at an elution volume of around 16 mL under these conditions.

### Cell Viability Studies

#### Cell Culture

Cell studies were carried out using two ovarian cancer cell lines (A2780, A2780cis) and a fibroblast cell line (MRC-5) from the CellBank Australia (Children’s Medical Research Institute, Sydney). The Roswell Park Memorial Institute 1640 (RPMI-1640) and Dulbecco’s Modified Eagle Medium (DMEM), each with 10% Fetal Bovine Serum (FBS), 1% GlutaMAX, and 1% penicillin/streptomycin were used as cell growth media. Cells were cultured on tissue culture flasks in a 5% CO_2_ incubator at 37 °C.

#### In Vitro Cell Viability

The in vitro cytotoxicity of the SCNPs was assessed on the A2780, A2780cis, and MRC-5 cell lines using the sulforhodamine B (SRB) assay. Cells were seeded into 96-well plates at a cell density of 4000 cells per well and incubated for 48 h at 37 °C and 5% CO_2_. Polymer solutions were UV-irradiated, and light-sensitive SCNPs and pure drug solutions were filtered for sterilization and diluted to various concentrations (polymers at 1 mg·mL^–1^; SCNPs diluted to 25 μM cisplatin) before being added to the cells.

After 72 h of incubation, the old medium was discarded and 100 μL of a cold 10% trichloroacetic acid (TCA) solution was added to each well for fixation, followed by a 30 min incubation at 4 °C. Thereafter, the cells were washed five times with 200 μL/well Milli-Q water, followed by adding 100 μL SRB in 1% acetic acid to each well. The plates were kept in the dark at room temperature for 20 min. Unbound dye was removed by washing the wells five times with 200 μL of 1% acetic acid and air-dried overnight in the dark at room temperature. 100 μL of 10 mM Tris buffer was added to each well and incubated for a minimum of 3 h prior to absorbance measurements at 570 nm using the Thermo Labsystems 354 Multiskan Ascent microplate reader (Thermo Fisher Scientific).

### Analysis Techniques

#### Conventional SEC and Hyphenation with Viscometry

Organic SEC on a Shimadzu modular system comprising a DGU-12A degasser, an LC-20A pump, a SIL-20AD autosampler, a CTO-20A column oven, and a RID-20A refractive index detector. For organic analysis, an 8 × 50 mm GRAM guard column and an 8 × 300 mm GRAM column (linear, 10 μm particle size, 500 Da −1 MDa, Agilent Technologies, Germany) were used for separation, while *N*, *N*′-dimethylacetamide (DMAc; HPLC grade) containing 0.25% w/v LiBr and 0.01% w/v BHT was used as an eluent. The system was operated at a flow rate of 1 mL·min^–1^ with the isocratic solvent mode at 50 °C in a CTO-20A oven. The injection volume was 50 μL. The system was calibrated using poly­(methyl methacrylate) calibration standards (PMMA, Agilent Technologies).

Aqueous SEC was implemented using an Agilent 1220 Infinity Compact LC system comprising an LC pump (G4281B), an autosampler (G4282B), a column oven (G4283A), a multiple wavelength detector (MWD, G4284B), and a diode array detector (Infinity 1260 DAD WR, G7115A). Another SEC system was implemented to use in hyphenation with a viscometer; a Shimadzu modular system comprising of DGU-12A degasser, LC-20A pump, SIL-20AD autosampler, CTO-20A column oven, SPD-20A UV/Vis detection unit, and RID-20A refractive index detector, in hyphenation with an online viscometer DVD-1260 (Agilent Technologies, Germany). An 8 × 50 mm SUPREMA guard column and an 8 × 300 mm SUPREMA column (linM, 5 μm particle size) were used for separation, while water containing 0.02% w/v NaN_3_ or 0.1 M disodium dihydrogen orthophosphate buffer (pH 8.7–9.3) was used as an eluent. The system was operated at a flow rate of 1 mL·min^–1^ with the isocratic solvent mode at 35 °C. The injection volume was 25–100 μL (depending on a detector setup). The system was calibrated using pullulan (Agilent Technologies, Germany) and polyethylene glycol/poly­(ethylene oxide) (PEG/PEO, Agilent Technologies) commercial standards. The detectors were calibrated using commercial pullulan and dextran standards (PSS Polymer Standards Service GmbH, Germany), the latter as a control for accuracy and robustness of the system.

### Nuclear Magnetic Resonance Spectroscopy

Nuclear magnetic resonance (NMR) spectroscopy was used to elucidate the structures of the obtained compounds and determine the reaction conversions. ^1^H NMR spectra were collected by a Bruker Advance III 400 MHz instrument equipped with an autosampler system. NMR spectra were processed by using TopSpin software. CDCl_3_ was used as the solvent.

### Dynamic Light Scattering

Dynamic light scattering (DLS) measurements were performed on a Malvern Zetasizer Nano ZS (He–Ne laser 633 nm, max 5 mW) with a light scattering angle of 173° (backscatter) and then analyzed using the Malvern Zetasizer software version 6.20. All samples were prepared in the desired solvent at various concentrations and filtered through a 0.45 μm pore size filter to remove dust prior to measurement. In this manuscript, the volume-weighted distributions have been reported.

### Inductively Coupled Plasma–Optical Emission Spectroscopy (ICP-OES)

ICP-OES was used to quantify the platinum content of the nanoparticles. SCNP solutions were digested under acidic conditions to remove any organic nonvolatile components. The analysis of digested samples was performed using an ICP-OES AVIO550 instrument from PerkinElmer.

Drug loading content was determined according to eq 1, as the weight of the polymer drug conjugates is the sum of the masses of cisplatin and polymer in the nanoparticles.^46^

The drug load efficiency was calculated according to eq 2, which is the amount of cisplatin attached to the polymer to the total amount of drug added in the feed.

Equation 1: Drug loading content of cisplatin via conjugation to polymers (as drug loading capacity (DLC) in percentage).

1 $$DLC\left(\%\right)=\frac{weight\;of\;cisplatin\;in\;the\;conjugate}{weight\;of\;PD\;conjugate}\cdot 100$$

Equation 2: Drug loading efficiency of cisplatin (DLE in percentage).

2 $$DLE\left(\%\right)=\frac{attached\;cisplatin}{total\;drug\;added}\cdot 100$$

### Small-Angle X-ray Scattering (SAXS)

Small-angle X-ray scattering (SAXS) measurements were made at the Australian Synchrotron’s SAXS/WAXS beamline using a 96-well plate autoloading system that injects the particle solution into the capillary. Isotropic scattering patterns were collected on a Pilatus 1 M detector (Dectris, Baden-Daettwil, Switzerland) using a wavelength of 1.033 Å (12 keV) and a sample-to-detector distance of 2.5 m, and the q range is around 0.005–0.3 Å^–1^. The measurement geometry was used to convert the counts per detector pixel into the radially averaged intensity of the scattering vector on an absolute intensity scale, and the average intensity was subtracted from the background of the scattering of the solvent in the capillary via the beamline software Scatterbrain. Data analysis and optimization of fitting parameters were performed in the SAS Data Analysis software ATSAS (EMBL Hamburg), plotting data in the double logarithmic (Supporting Information, Figure S1) and Guinier plot. To determine the *R*_g_ values, the software tool for *R*_g_ analysis (Primus Guinier Plot) and the dimensional distribution analysis was used to fit the scattering data, using the fitting model for a global or arbitrary monodisperse structure as one of the built-in options.

## Results and Discussion

### Polymer Library

The polymer library used herein is a series of statistical copolymers, P­(PEGMEA_*x*_-*co*-CEA_*y*_), with and without the incorporation of hydrophobic monomers, synthesized via reversible addition–fragmentation chain-transfer (RAFT) polymerization of PEGMEA ( ${\mathrm{M̅}}_{n}$ = 480 g·mol^–1^) and CEA. Polymers vary in chain length (target degree of polymerization (DP) of 100 and 200) and monomer composition (CEA 20–80 mol %). This type of statistical copolymer was chosen for its high water solubility, attributed to the PEG chains and the evenly distributed carboxylic groups along the polymer chain, which serve as cross-linking points.

The second set of polymers, P­(PEGMEA_*x*_-*co*-CEA_*y*_-*co*-MA/BA/BzA_*z*_), was prepared containing hydrophobic repeating units (Scheme 1). The three different hydrophobic monomers (MA, BA, and BzA), differing in hydrophobicity and structure, were tested at 10–40 mol %, screening for the highest compaction without compromising the water solubility of the resulting SCNP. PEGMEA and CEA were used as hydrophilic components at a fixed molar ratio of [50:50], allowing the SCNPs to stay water-soluble up to a specific monomer composition, beyond which the polymer chains begin to increasingly form aggregates. Morishima et al.^54^ reported that hydrophobic moieties can promote the self-association of polymer chains in an aqueous environment. The copolymers were prepared using RAFT polymerization in a similar manner, and the results are summarized in Table 1. The names of the P­(PEGMEA_*x*_-*co*-CEA_*y*_) polymers are derived from the target DP and the mol percentage of CEA, which is coined here as cross-linking points (CLPs).

Conversion of the monomers was determined using the differences between the integrals for the vinyl peaks assigned to each monomer (δ 6.25–6.4 ppm) when taking an NMR spectrum before and after the reaction. After determining the incorporation rates of CEA and PEGMEA during homopolymerization, it was concluded that both acrylate monomers reached similar conversions at certain times, with negligible differences (Supporting Information, Figure S2), suggesting reactivity ratios close to 1.

Based on the conversion of the monomers determined via ^1^H NMR, a theoretical molecular weight $\left({\mathrm{M̅}}_{n,theo.}\right)$ was calculated for the obtained copolymers. For the following experimental steps with the polymer precursors, ${\mathrm{M̅}}_{n,theo.}$ was used due to its higher reliability than the collected ${\mathrm{M̅}}_{n}$ from the SEC in DMF. The notable difference between ${\mathrm{M̅}}_{n,theo.}$ and ${\mathrm{M̅}}_{n,\mathrm{SEC}}$ arises from differences in the swelling behavior of the random copolymers compared to the applied SEC calibration standards (i.e., poly­(methyl methacrylate), PMMA).

Prior to the formation of SCNPs and CDDP attachment, the water solubility of these polymers must be ensured. The polymer–drug conjugation were carried out in water at a slightly alkaline pH (pH 8.7–9.3) to ensure full deprotonation of the COOH groups. The dissolution into a single polymer chain for all polymers was confirmed with DLS, which also shows a larger hydrodynamic diameter at pH 8.7–9.3 due to full deprotonation and subsequent repulsion of the negative charge (Supporting Information, Figure S3).

The polymers were characterized using SEC in DMF and in water. Aqueous SEC with amphiphilic polymers requires method optimization, as these polymers are prone to column interactions. An aqueous mobile phase containing 0.02 wt % sodium azide as the mobile phase was chosen to start with, performing measurements on the SUPREMA linM 5 μm column. No significant changes in the elution profiles were revealed for samples with increasing hydrophobicity of P­(PEGMEA-*co*-CEA-*co*-MA/BA/BzA) (Supporting Information, Figure S4). The temperature increase reduces the viscosity and the interactions between the polymer and the column material, providing insights into whether enthalpy is involved. The robustness of the system was demonstrated by repeating the measurements at an elevated temperature (i.e., 45 °C), with no significant changes in the elution profiles of the polymer; therefore, strong secondary interactions between the sample and the column material could be ruled out. The ionic strength of the mobile phase was then carefully increased to 0.1 M disodium dihydrogen orthophosphate buffer at a pH 8.7–9.3 to shield the charges of the deprotonated COOH while preventing chain expansion and aggregation. Based on the results, the SEC method with 0.1 M phosphate buffer appeared to better represent the polymer chains in their dissociated state than in 0.2 g·L^–1^ NaN_3_. Contrary to expectations that 30 mol % BzA elutes prior to 30 mol % BA and MA based on ${\mathrm{M̅}}_{n}$ _,theo._, it elutes after 30 mol % MA in the 0.1 M PB and at a similar elution volume as 30 mol % BA. This result implies either (i) a greater collapse of the BzA polymer chain into a more compact coil due to its higher hydrophobicity or (ii) an additional interaction with the stationary phase. To investigate the precollapse behavior of the amphiphilic copolymers, SEC-viscometry was performed (Figure 1). The comparison of signal intensities of the specific viscosity obtained for 30 mol % MA, BA, and BzA clearly shows a significant difference in differential pressure (Δ*p*) recorded at the membrane of the viscometer; 30 mol % MA shows a higher Δ*p*, indicating a more viscous sample than BA and BzA. After the extrapolation to infinite dilution (intrinsic viscosity), which is achieved by a large volume reservoir within the detector, the intrinsic viscosity decreases with the increase in the logP value of the applied hydrophobic comonomer (logP: MA < BA < BzA, Figure 1b). As depicted in Figure 1b, polymers with a lower degree of hydrophobicity, either due to the amount of hydrophobic comonomers or the logP value, showed a much larger drop in the intrinsic viscosity when dissociated in 0.1 M PB, confirming the results obtained from the conventional SEC (Supporting Information, Figure S4). The insights gained on the effect of the hydrophobicity on our precursor material raised the question of the extent to which the chain self-association contributes to the compaction of SCNPs made from these polymers, and whether there is a limit at which the system becomes too hydrophobic when cisplatin is added to the polymers, or it becomes too sterically hindered to maintain compaction and could potentially undergo intermolecular cross-linking or aggregation. Earlier papers have shown that the preorganization of the polymer prior to cross-linking influences the final structure.^10,59^ Preorganization in a suboptimal structure can be detrimental to the properties,^59^ but knowledge of the relationship between preorganization and cross-linking can also aid the outcome.^15^

**Figure 1:**
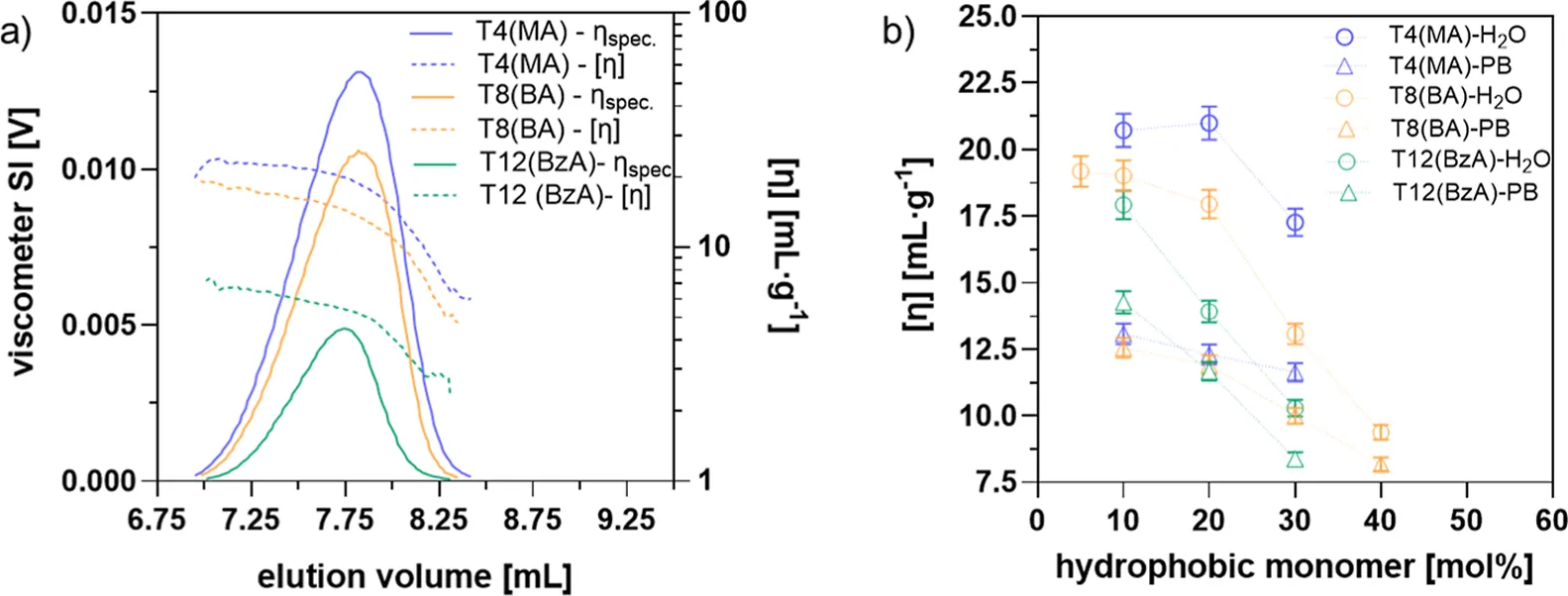
Comparison of SEC-viscometry chromatograms of polymer samples differing in the type of incorporated hydrophobic monomer (i.e., MA, BA, BzA). (a) With increasing degree of hydrophobicity (logP), the specific viscosity, signal intensity, and thus the intrinsic viscosity drop (i.e., logP 0.8 (MA), 2.3 (BA), and 2.6 (BzA)). (b) Correlation between the intrinsic viscosity and the degree of hydrophobicity of the polymer samples in two different solvents (i.e.,H_2_O + 0.02 wt % NaN_3_, 0.1 M phosphate buffer), which differ in a hydrophobic comonomer content and logP values.

### Conjugation of Platinum Drugs

The synthesized statistical copolymers were used as a precursor material for the conjugation of cisplatin with the carboxylic groups after their water-solubility was confirmed by DLS (Supporting Information, Figure S3) and SEC (Supporting Information, Figure S4,S5). The initial conformation in solution before cross-linking will influence the compactness of the final product (Scheme 2).^60^ To reduce the interchain surface contact between multiple polymer chains during the cross-linking reaction, the conjugation process needs to be carried out in highly diluted solutions.^61^ Hence, the herein-described polymer-drug conjugation with cisplatin was performed at a polymer concentration of 0.4 g·L^–1^. Two different feed ratios of cisplatin, [1:2] (leading to PD1) and [1:1] (leading to PD2) cisplatin to COOH groups, were tested with regard to the mechanism of polymer–drug conjugation. A ratio of [1:2] was assumed to be the ideal state in which one drug molecule cross-links between two pendant chains on the polymer, thus an individual polymer chain collapses intramolecularly to form an SCNP. The [1:1] feed ratio was assumed to have fewer cross-linked COOH groups, thereby reducing the likelihood of cross-linking and increasing the probability of pendant cisplatin (Scheme 2). The polymer was dissolved in a dilute NaOH solution to ensure deprotonation. The resulting solution had a pH of around 8, which helped with full solubility without damaging the ester structure. The addition of the CDDP solution and dilution with water to a final concentration of 0.4 g·L^–1^ resulted in a neutral solution. The mixture was left to react for 8 h at 37 °C in a water bath. The rate of reaction was initially evaluated using SEC-UV/Vis analysis at a detector wavelength of 290 nm (Supporting Information, Figures S6). Reaction samples were probed at the start and end of the reaction time (i.e., 0 h, 8 h). The differential peak area of the peak associated with unbound free cisplatin was assigned to the consumption of free cisplatin, thus representing the percentage of cisplatin conjugated to the polymer over time. After removing the excess amount of unbound cisplatin via dialysis, the amount of conjugated cisplatin after 8 h was determined via ICP-OES (Supporting Information, Tables S1 and 1).

**Scheme 2:**
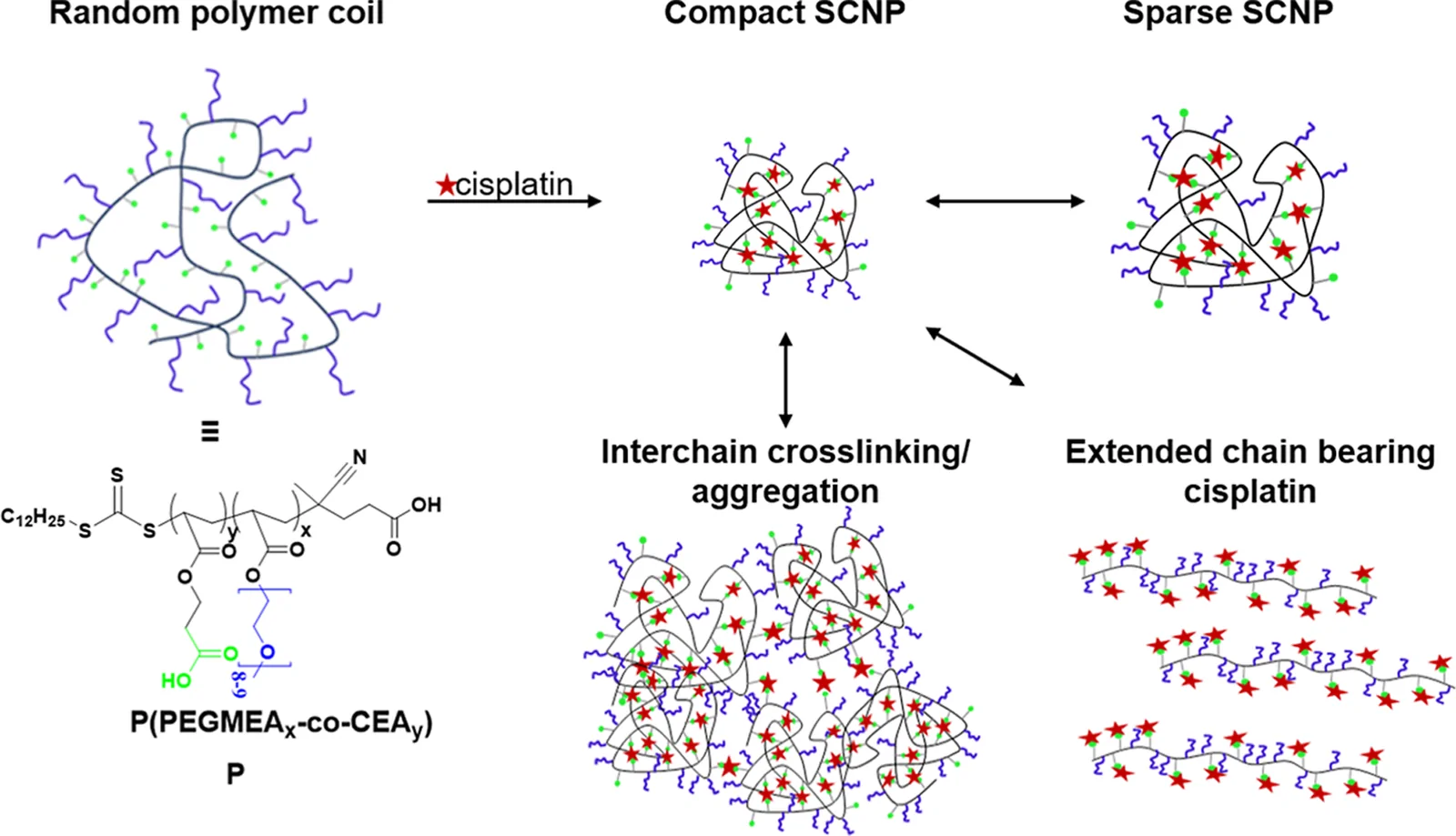
Conjugation of Cisplatin to Polymer Chains with Various Events of Outcomes: Intramolecular Cross-linking into Compact or Less Compact/Sparse SCNPs, Interchain Cross-linking or Aggregation, or Lose Extended Polymer Chains Bearing Cisplatin on Pendant Chains

After reacting CDDP with the COOH groups at a molar ratio of [1:2] (PD1 series), approximately 50–70% of the CDDP has reacted, which can be expressed by drug loading efficiency (DLE), leaving about 30% of the COOH groups unreacted (Supporting Information, Table S1). The results are largely independent of the total number of RUs, but there is a subtle decline with an increasing CEA content, which is not surprising, given that a large amount of CEA forms a denser network with restricted movement. Increasing the CDDP concentration in the reaction mixture to a [1:1] ratio (PD2 series) further saturates the remaining COOH groups, but the overall DLE decreases. As the DLE is only slightly affected by the CEA, the amount of conjugated drugs, expressed as drug loading capacity (DLC), increases almost linearly with the COOH content (Figure 2). The COOH/CDDP feed ratio of [1:1] was used for the amphiphilic polymers, resulting in a significantly higher percentage of conjugation. The hydrophobic pockets may have helped to retain the slightly hydrophobic CDDP within the SCNP, facilitating the conjugation reaction.

**Figure 2:**
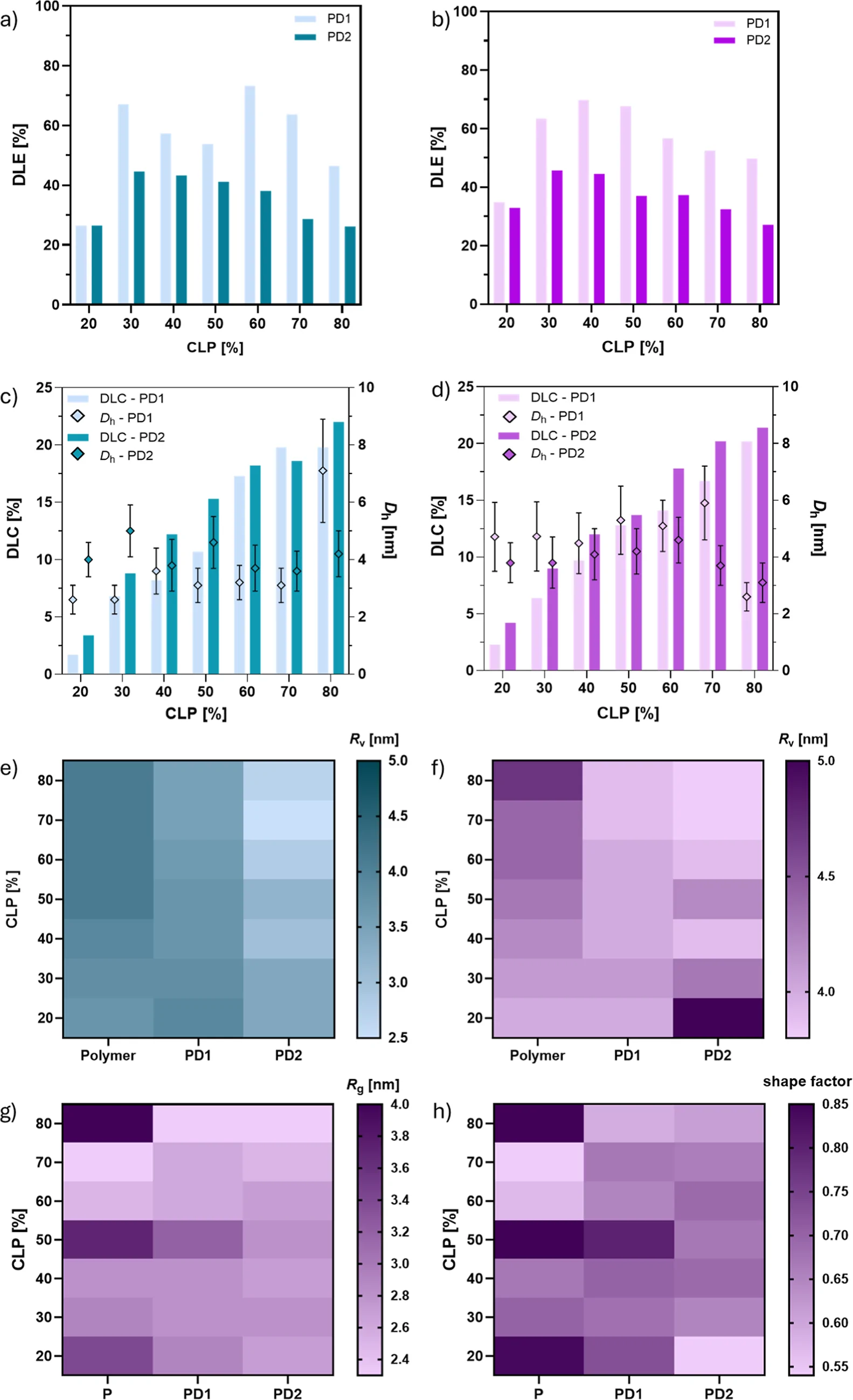
(a,b) Drug loading efficiency (DLE) of copolymers of target DP 100 (a) and DP 200 (b) as a function of increasing cross-linking points (CLP % ∼COOH) along the polymer chains. (c,d) Correlation between cross-linking points (CLPs) along the polymer chain of target DP 100 (c) and DP 200 (d), the drug loading content of cisplatin, and the hydrodynamic diameter determined via DLS. PD1 and PD2 series show SCNPs resulting from a [1:2] and [1:1] cisplatin-to-CLP feed ratio, respectively. DLC was determined via ICP-OES; MDL-method detection limit at 0.05 ppm; this is the lowest concentration at which the analyte can be detected in a sample with 99% certainty. (e,f) Comparison of *R*_v_ of polymer and polymer–drug conjugates (PD1, PD2) for target DP 100 (e) and DP 200 (f). (g) *R*_g_ from SAXS and resulting shape factors of DP 200 polymer precursors and their polymer–drug conjugates (PD1, PD2) varying in cross-linking points and polymer–drug feed ratios. (h) Shape factors are approximated based on *R*_g_/*R*_v_.

### Dimensional Changes upon Platinum Drug Conjugation

Since cisplatin was used both as a drug and as a cross-linker, it raises the question of how drug loading with cisplatin influences the compaction of the SCNPs. To assess the effects of these factors, techniques such as dynamic light scattering (DLS), size exclusion chromatography (SEC), SEC-viscometry, and SAXS (small-angle X-ray scattering) were employed to characterize the P­(PEGMEA_*x*_-*co*-CEA_*y*_) series initially, and the reliability of these analytical methods is discussed here.

To assess dimensional changes from a linear extended polymer chain to a denser structure upon cross-linking, our first analytical approach was DLS in order to compare hydrodynamic radii (*R*_h_) before and after the conjugation reaction. DLS was selected first because it is an easily accessible, quick benchtop laboratory solution for NP characterization. Polymer–drug conjugates were analyzed in water at pH 7 after dialysis, as changes in osmotic pressure or in pH to acidic values would have induced drug release. At pH 7 in pure water, there was no measurable drug release during the analysis period. The hydrodynamic diameter was measured to be around 3–6 nm, with longer polymers resulting in slightly larger SCNPs (Figure 2c,d). Due to limitations of the detector sensitivity in the sub-10 nm size range and using a fitting model of sphere-like particles, polymeric samples and SCNPs are challenging to investigate via DLS. DLS can confirm the absence of intermolecularly cross-linked polymers (Scheme 2), but it is not suitable for finer details.

A better approach for analyzing SCNPs destined for drug delivery is aqueous SEC. Conventional SEC–here defined as a SEC system hyphenated with concentration detectors (i.e., UV/Vis, RI) only, enables monitoring the event of compaction of a single linear polymer chain as a function of a decrease in the hydrodynamic volume; a shift to higher elution volumes was expected.^62^ Among all statistical copolymers P­(PEGMEA_*x*_-*co*-CEA_*y*_), which vary in the number of cross-linking points (%CEA), chain length, and degree of PEGylation, a shift to larger elution volumes was observed (Supporting Information, Figure S5). The decrease in *V*_h_ can be attributed to increased compactness; however, the elution behavior can also be affected by electrostatic interactions between the samples and the stationary phase, as the addition of the cross-linker (i.e., drug) increased hydrophobicity.

Samples with only 20 mol % CEA (i.e., 100-20 and 200-20) showed the least increase in elution volume, which could be either due to the lowest number of available cross-linking points (%CEA) or as well due to the lowest amount of cross-linkers attached, thus the lowest addition of hydrophobic drug with 3.4 and 4.2 wt % for 100-20-PD2 and 200-20-PD2, respectively. The comparison of the drug amount and relative shifts in elution volumes between samples does not show a clear correlation in conventional SEC. However, in an aqueous environment, we assume that the cross-linked polymer chain will arrange itself with a hydrophobic core and PEG chains grafted onto the NP shell.^58^ While the conventional SEC provides only qualitative information on the degree of compactness, we therefore hyphenated the SEC with a viscosity detector to enable deeper insights into the structural and dimensional changes during polymer–drug conjugation.

The viscosity detector provides intrinsic viscosity as its primary output, based on changes in differential pressure that reflect sample density and conformation. To obtain the intrinsic viscosity, the software requires the differential pressure from the viscometer and the injected mass of a fully recovered sample based on the signal from the concentration detector (i.e., RI detector). Hence, the obtained intrinsic viscosity is solely dependent on the full recovery of the sample, e.g., shifts in elution volume due to interactions do not affect the intrinsic viscosity values, but a lack in recovery of the injected sample is impacting the calculations of the injected mass and leads to false viscosity data. As secondary information, the molar mass can be determined via a universal calibration. However, a reliable and robust universal calibration and data set is solely obtained if an interaction-free system is given, as log­([η]·M) is plotted against the elution volume.

Since intrinsic viscosity is inversely proportional to molecular density, viscosity measurements enabled us to determine the overall density of the nanoparticles and to observe changes in their compactness after cross-linking. Doubling the chain length of the polymer backbone yields particles with slightly higher intrinsic viscosities (Supporting Information, Figure S6a) and higher levels of conjugated CDDP (PD2 series), leading to greater compaction (Supporting Information, Figure S6b) as more hydrophilic COOH groups are modified with drugs. Compared with proteins, e.g., bovine serum albumin (BSA), which was found to have an intrinsic viscosity of 3.1–3.8 mL·g^–1^ and a hydrodynamic diameter of approximately 7 nm,^63^ allows for a further understanding of how far the SCNPs are away from a globular, hard-sphere-like structure such as a protein. Therefore, we hypothesize that our system of a hydrophilic polymer precursor collapses intramolecularly based on two effects at the same time: cisplatin promotes self-associated folding by introducing hydrophobicity in the copolymer chain as being attached to one of the pendant chains^54^ and working as a cross-linker between two CEA pendant chains. Hence, the compaction, driven by two effects, presumably forms a dense, hydrophobic core of cisplatin with PEG chains facing outward.

An additional feature of viscosity measurements is the deduction of the hydrodynamic volume *V*_h_ from the intrinsic viscosity, which can be further used to access the hydrodynamic radius via the Einstein–Simha equation *V*_h_ = *M*[η]/(2.5 N_A_),^32,50,64^ where *V*_h_ is the hydrodynamic volume [cm^3^], *M* is the molar mass [g·mol^–1^], [η] is the intrinsic viscosity [mL·g^–1^], and *N*_A_ is the Avogadro constant; 6.022·10^23^ mol^–1^. From there, the viscosity radius *R*_v_ can be calculated using *R*_v_= (3*V*_h_/4π)^1/3^ (Figure 2e,f). The viscosity diameter is an important parameter when other size-determining techniques reach their limits (e.g., MALS, DLS).^65^

The significant difference between radii obtained by viscometry (*R*_v_) and DLS under similar conditions (i.e., water) (Supporting Information, Table S2) can be attributed to fundamentally different approaches used to determine the hydrodynamic radius. In both approaches, a spherical model is applied. However, the raw data acquired in online viscometry is the differential pressure (a physical parameter), which is subsequently related to the samples’ intrinsic viscosity without any underlying model or approximation (absolute data). Only for the following step, when the radius is derived from the viscosity (*R*_v_), the hydrodynamic volume-to-radius conversion for a sphere-like particle is used. The *R*_v_ depicts a more realistic approximation of the polymer chains in solution as random coils than the comparable data set from DLS measurements in water. Nevertheless, *R*_h_ and *R*_*v*_ should be subjected to criticism regardless of the chosen method, also due to the samples’ polydispersity and the consequent effect on the radius calculations, as it does not provide information on how far the SCNP is from a globular state. Therefore, to determine the nanoparticle shape and transition stage, the shape factor ρ provides deeper insights into the morphology of the SCNPs.

The shape factor ρ is defined as a relation between the radius of gyration *R*_g_ and the hydrodynamic radius *R*_h_. It therefore helps to compare the segmental density of the non-cross-linked and cross-linked structures and to support the hypothesis regarding which transition state the SCNPs are in. Values around 0.78 are associated with proteins and hard spheres, while 1.50 represents a random coil structure. If the shape factor exceeds 1.5, the sample may, for example, adopt a rod-like structure.^59,66^ A reliable technique for determining the radius of gyration (*R*_g_) of these sub-10 nm particles is small-angle X-ray scattering (SAXS). Where MALS detection reaches its limit of detection and sensitivity (i.e., D < λ/20 for an isotropic scatterer), SAXS fills the gap and serves as a powerful analytical tool for the characterization of very small architectures. SAXS experiments were carried out using the DP 200 polymer precursors and the corresponding PD1 and PD2 nanoparticle series in water at pH 7 (Figure 2g). With *R*_g_ values from SAXS and *R*_h_ either from DLS in water or the Einstein–Simha approximation, the shape factor ρ (*R*_g_/*R*_h_) can be calculated (Table 2).^65^ It is evident that the shape factors ρ derived from DLS data are nonsensical, further underlining that DLS may not be a suitable technique for SCNPs (Supporting Information, Table S2) and therefore only the viscosity radius was used (Figure 2 h). However, many values obtained are below 0.78, the value for a hard sphere, which is, in theory, the lowest possible value. One reason is that *R*_v_ equals *R*_h_ only under specific circumstances, and deviations from the assumed spherical shape, especially when measuring compact molecules such as dendrimers, cause *R*_v_ to be lower than *R*_h_.^67,68^ In our case, a certain degree of heterogeneity in size, structure, and dimension of the samples needs to be considered, which further complicates the interpretation of the shape factor. The shape factor is therefore only a rough guide, subject to uncertainties as discussed.

**Table 2:** Comparison of the Shape Factor for SCNPs of Target DP 200 Using *R*_g_ via SAXS, *R*_h_ from DLS, and *R*_v_ from Viscometry/Einstein–Simha Approximation

polymer	CLP %	*R*_g_ ^a^ [nm]	*R*_h_ ^b^ [nm]	*R*_v_ ^c^ [nm]	ρ^b^	ρ^c^
200-20	20	3.4	1.6	4.0	2.10	0.84
200-30	30	2.9	1.3	4.1	2.20	0.70
200-40	40	2.8	1.5	4.2	1.86	0.66
200-50	50	3.7	1.8	4.3	2.03	0.85
200-60	60	2.5	1.0	4.4	2.51	0.57
200-70	70	2.3	1.0	4.4	2.34	0.53
200-80	80	4.0	1.0	4.7	3.99	0.85

a Determined via SAXS.

b Determined via DLS.

c Via Einstein–Simha approximation of viscosity data. ρ^b^: shape factor applying *R*_g_^a^/*R*_h_^b^. ρ^c^: shape factor applying *R*_g_^a^/*R*_v_^c^.

Alternatively, the intramolecular cross-linking occurring within one polymer chain can be approximated by the contraction factor g’, which is a measure of a polymer’s compactness based on the ratio between the intrinsic viscosity of the long-chain-branched polymer and that of a linear polymer. In our case, we can apply this by treating our SCNPs as contracted polymers and defining the polymer precursor as linear. The contraction of the hydrophilic polymer chains within the SCNPs increases with the number of cross-linking points and could therefore help quantify the degree of compactness. For the PD2 series, a molar drug to COOH feed ratio of [1:1] shows, for both polymer chain lengths, lower g’ factors but a similar trend to the PD1 series (Figure 3a).

**Figure 3:**
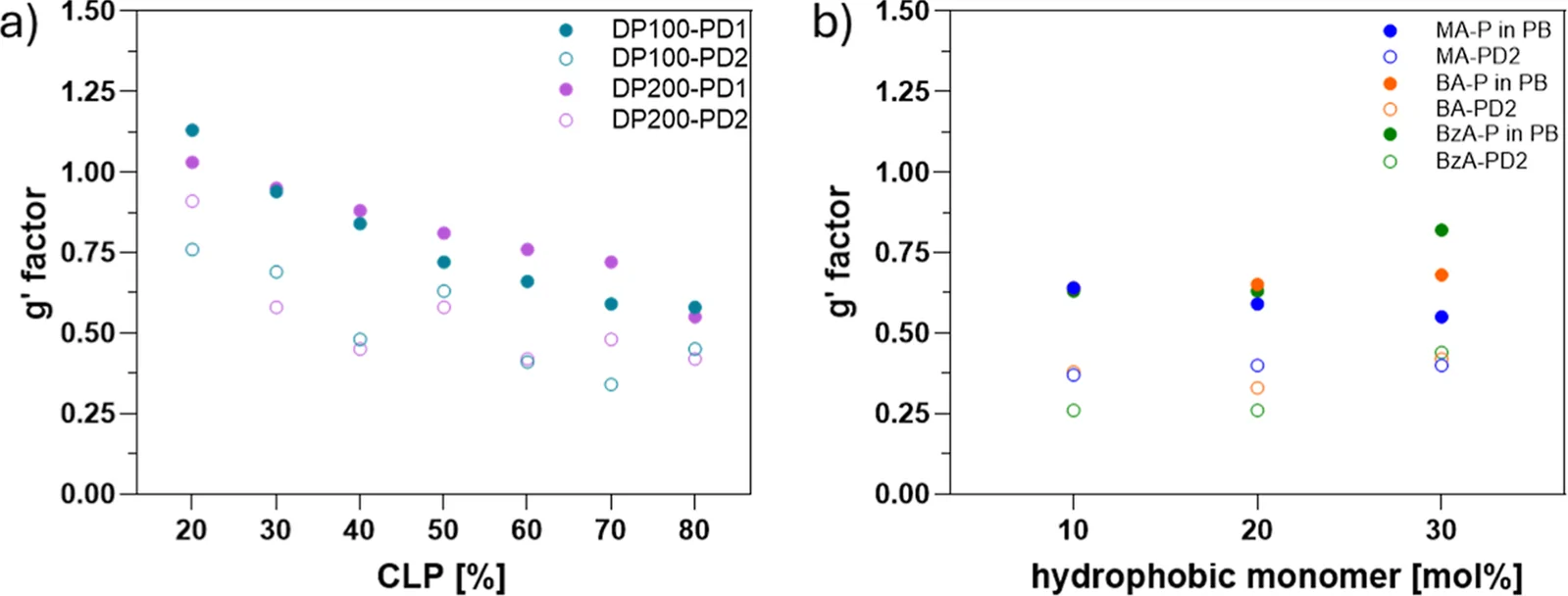
Contraction factor g’ as a function (a) of the amount of cross-linking points (%CLP–% of COOH) and precursor chain length (DP 100, DP 200) for the PD1 and PD2 series of the SCNPs in water and (b) of the degree of hydrophobicity in the polymer backbone (i.e., 10–30 mol % MA, BA, or BzA); comparing the contraction efficiency of precursor dissolved in 0.1 M phosphate buffer at pH 8.7–9.3 (MA/BA/BzA-P) with the SCNPs in water (MA/BA/BzA-PD2).

Despite some uncertainties in the shape factor, trends can be extracted from Figure 2 and Figure 3a. A drug-to-cross-linker point feed ratio of [1:1] (PD2 series) was found to generate SCNPs with higher compaction than with other feed ratios. Results from conventional SEC and SEC-viscometry indicated that the polymer chains adopted a more compact conformation upon conjugation with CDDP. SAXS analysis allows the determination of *R*_g_ and thus the shape factor, especially when nanoparticle sizes fall below the threshold for anisotropic scattering and MALS is not possible.

We then sought to determine whether the addition of hydrophobic repeating units could enhance compactness. After noting that higher hydrophobicity promotes self-association within a single polymer chain prior to CDDP conjugation, we aimed to assess how this increased compactness influences SCNPs’ formation. The drug-conjugated amphiphilic SCNPs were characterized primarily by SEC, following DLS analysis that confirmed the absence of particles >10 nm. The SEC elution diagrams showed a clear shift to larger elution volumes, indicating a reduction in hydrodynamic volume. On the basis of the results, it seemed that the precursor containing 30 mol % BA experienced the strongest intramolecular collapse as it shifted the most and eluted at larger elution volumes than 30 mol % MA and BzA (Figure 4d).

**Figure 4:**
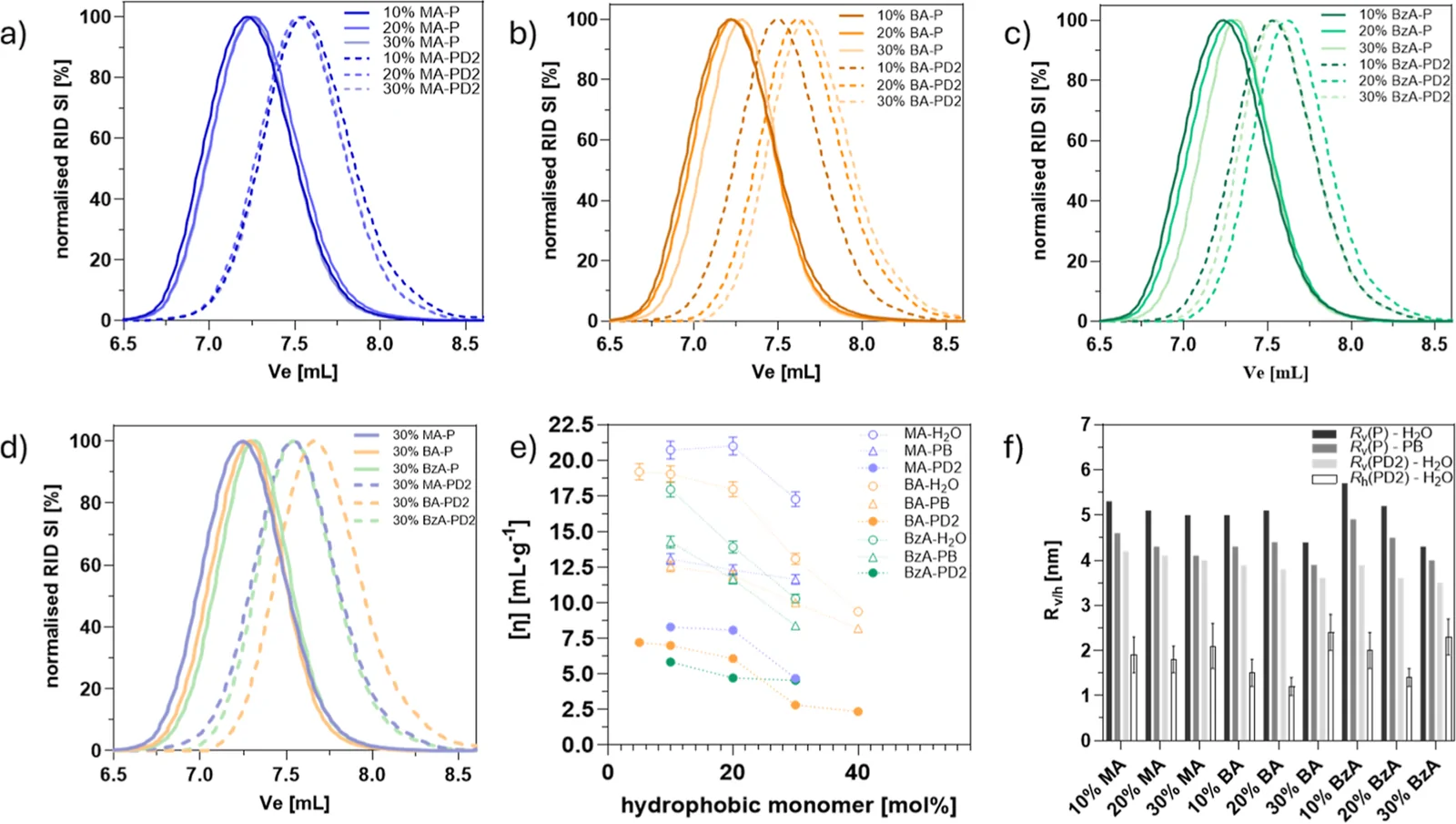
(a–d) Peak shift in elution volume upon drug conjugation monitored via SEC-RI. (a) For P­(PEGMEA-*co*-CEA-*co*-MA) from 10–30 mol % MA, (b) for P­(PEGMEA-*co*-CEA-*co*-BA) from 10 to 30 mol % BA, and (c) for P­(PEGMEA-*co*-CEA-*co*-BzA) from 10 to 30 mol % BzA. (d) Overlaid chromatograms of copolymers and SCNPs of 30 mol % MA, BA, and BzA comparing the effect of different degrees in hydrophobicity on SEC elution profiles. (e) Correlation between the intrinsic viscosity and the degree of hydrophobicity of the polymer and SCNPs, which vary in a hydrophobic comonomer composition and thus logP values. MA = methyl acrylate, logP 0.8; BA = butyl acrylate, logP 2.3; and BzA = benzyl acrylate, logP 2.6. (f) Comparison of radii derived from viscometry and DLS for the copolymers in water, copolymers in buffer (pH 8.7–9.3), and SCNPs in water.

Again, SEC-viscometry was applied to obtain absolute data on compactness, independent of potential interactions within the SEC system, provided that there was full sample recovery. Both tested mobile phases, water (0.02 wt % NaN_3_) and 0.1 M phosphate buffer at pH 8.7–9.3, show a significant reduction in intrinsic viscosity and, thus, an increase in compaction upon conjugation with cisplatin (Figures 3b and 4e). Interestingly, the SCNP containing 30 mol % BA exhibits the highest compactness among those with equivalent molar ratios of hydrophobic comonomers, consistent with results from conventional SEC. It is likely that the slightly higher steric hindrance of the benzene ring provides less chain flexibility than that of a butyl group. This concludes that hydrophobicity promotes compaction and is therefore beneficial for more compact NP systems up to a certain amount. Once the polymer chains become too hydrophobic, they can prevent reorganization into the most compact SCNPs.^69,70^

Even with the reduction in chain expansion due to electrostatic shielding, the drop in density from precursor chains to SCNPs upon cross-linking remains significant (Figure 4e). To better understand the significance of the difference in density, Einstein–Simha’s approximate radius calculation better illustrates the extent to which the dimensional changes occur. While polymers in water have a significantly larger radius due to chain expansion, the actual size difference between copolymers in phosphate buffer and SCNPs in water varies from sample to sample; however, it is not as drastic. This confirms that the chain collapses into a more compact, folded structure, driven by intramolecular cross-linking by cisplatin, which contributes to a slight increase in hydrophobicity.

### In Vitro Evaluation

Prior to cytotoxicity testing, drug release under conditions representative of the intracellular environment was assessed. The SCNPs were placed in PBS, and the release was monitored using SEC (Supporting Information, Figures S7,S8) by the gradual increase of the area under the peak of released cisplatin at around 16 mL between 0 and 108 h (Figure 5b,d). The drug was fully released after 72 h, which is the time frame of the planned toxicity tests. Furthermore, the slight shift in elution volumes of the SCNPs toward smaller Ve indicates a reverse mechanism of intramolecular cross-linking, from compact SCNPs back to a polymer coil (Figure 5a,c). The final distribution shows only the polymer species and the free drug; other signals, such as those for potentially cleaved PEG, are absent, confirming the stability of the polymer during the release. Occasionally, such as in the case of the polymer example shown in Figure 5a, there is a small shoulder at higher molecular weights that might indicate some permanent cross-linking, although this byproduct formation is usually not observed.

**Figure 5:**
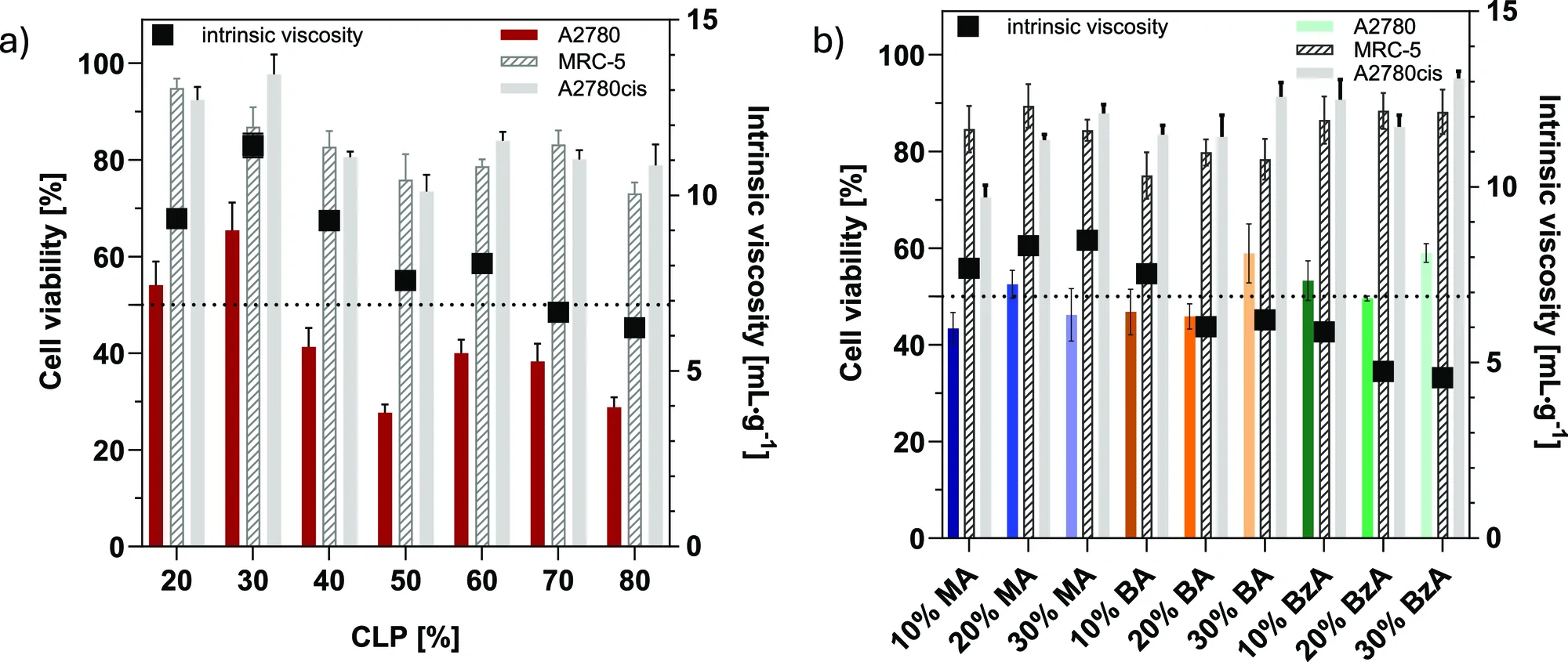
Drug release studies of Pt-SCNPs. (a, c) SEC profile of SCNPs 200-60-PD2 and T12-PD2, respectively, at different time points (i.e., 0–108 h), shifting from larger to smaller *V*_e_ as a function of drug release and therefore the reversing of intrachain cross-linking. (b, d) Increase in signal intensity of cisplatin over time for sample 200-60-PD2 and T12-PD2, respectively (i.e., 0–108 h).

Moreover, the toxicity of the polymers prior to CDDP conjugation was assessed using the ovarian cancer line A2780, as ovarian cancer is commonly treated with cisplatin. The polymers did not inhibit proliferation at a relatively high concentration of 1 mg·mL^–1^, which is well above the concentration used for cytotoxicity testing of the platinum drug-containing SCNP (Supporting Information, Figure S9). The aim was to examine whether cytotoxicity differed with variations in size, compaction, or hydrophobicity, as potential drivers of their biological activity. To enable comparability across SCNPs that differed in the amount of loaded drugs, the cisplatin concentration in the SCNP solutions was fixed for each test. Cells were incubated with SCNP solutions containing 25 μM platinum for 72 h.

At 25 μM cisplatin, a trend in cytotoxic behavior became visible for the P­(PEGMEA-*co*-CEA) SCNP series (Figure 6a): cytotoxicity increased with COOH content, and thus with the amount of drug per particle. Similar trends were observed for A2780 and the fibroblast cell line MRC-5. In contrast, no clear trend was observed in the amphiphilic NP series (Figure 6b), except that hydrophobicity reduced cytotoxicity. As both SCNP series exhibited sizes below 10 nm, no significant size difference could account for the clear difference in cytotoxic behavior of the amphiphilic SCNPs. In addition to size, another physicochemical parameter to consider that may influence differences in cytotoxic behavior is the compaction of the SCNPs. As shown in Figure 6a, the cytotoxicity of the SCNPs from the P­(PEGMEA-*co*-CEA) precursor appears to correlate with the reduction in intrinsic viscosity and thus with the increase in the particle compaction, which contrasts that of the hydrophobic series. Once hydrophobic comonomers are present in the SCNPs, they effectively reduce cytotoxicity despite greater compaction. Therefore, we hypothesized three possible scenarios occurring with the amphiphilic SCNPs that need to be investigated: (i) enhanced protein interactions, (ii) longer retention time in the cell membrane due to enhanced hydrophobic interactions with the lipids, and (iii) differences in drug release due to different unfolding of the chains and longer retention of the hydrophobic drug the higher the hydrophobicity of the copolymers. All three scenarios will be investigated in more in-depth and addressed in our next paper. It appears most likely that scenario (i) is not likely to occur, as previous studies on protein corona formation via DLS showed no significant changes upon incubation of SCNPs with FBS.^10^

**Figure 6:**
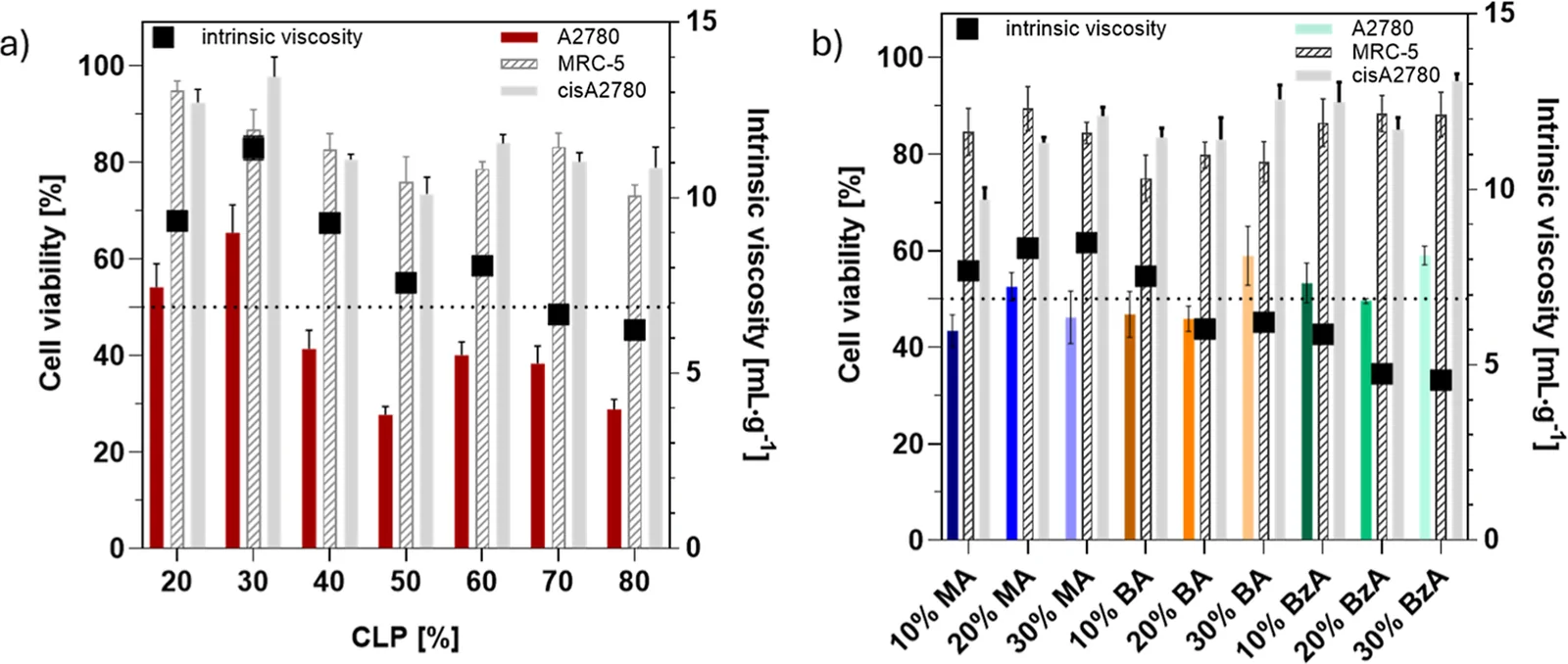
Cytotoxicity assessment of cisplatin-SCNPs on ovarian cancer cells A2780, healthy lung cells MRC-5, and cisplatin resistant ovarian cancer cells A2780cis at a cisplatin concentration set to 25 μM. (a) Comparison of cytotoxicity of SCNPs based on P­(PEGMEA-*co*-CEA) at varying cross-linker points (CLPs = %CEA). (b) Cytotoxic profile of SCNPs based on P­(PEGMEA-*co*-CEA-*co*-MA/BA/BzA) varying in mol % of hydrophobic moieties in the polymer and type of hydrophobic monomer.

## Conclusions

A series of P­(PEGMEA-*co*-CEA) statistical copolymers were prepared, which were used to conjugate cisplatin, forming SCNPs. The resulting SCNPs were water-soluble and could be characterized in water. With increasing CEA content, and therefore increasing Pt-based cross-linker content, the SCNP became increasingly compact, as evidenced by the lower intrinsic viscosity and smaller radius of gyration. Higher compactness is directly associated with increased cytotoxicity toward ovarian cancer cells. It is likely that this is due to the improved cellular uptake, but future detailed studies will need to evaluate the mechanism of translocation and quantify the cellular uptake. As chain compaction appeared to enhance cytotoxicity, we incorporated hydrophobic comonomers into the polymer to further promote intramolecular interactions. We hypothesized that increased hydrophobicity, which was achieved by copolymerizing MA, Ba, or BzA, would improve compactness and therefore enhance cellular uptake to deliver more drugs. However, this was not the case, and future investigations need to evaluate whether the presence of the hydrophobic copolymers hinders cell uptake or drug release.

## Supplementary Material



## Data Availability

The raw data is available from the authors upon request.

## Notes

Kinetic data on the copolymerization, SEC and DLS data to tests water solubility, DLC and DLE of the platinum drug conjugation, SEC diagrams in water after platinum drug conjugation, correlation of drug loading content of cisplatin and the intrinsic viscosity, details on the drug release study, and cell viability of the polymers without attached drug (PDF)
