# Supplementary material for: Engineering Intramolecular Compactness in Water-Soluble Single-Chain Nanoparticles for Optimized Platinum Delivery
Source: ACS Polym Au. 2026 Jun 24;6(5):1339–53. doi: 10.1021/acspolymersau.6c00083 (PMC13673886; doi:10.1021/acspolymersau.6c00083)
Supplement: Supplementary file 1 [file lg6c00083_si_001.pdf]

SUPPORTING INFORMATION

Engineering Intramolecular Compactness in water-soluble Single-Chain Nanoparticles for Optimized Platinum Delivery

Miriam B. Lossa,[a] Thorsten Hofe,[b*] Martina H. Stenzel[a*]

a) School of Chemistry, University of New South Wales, Sydney NSW 2052, Australia

b) Agilent Technologies Deutschland GmbH, 51120 Mainz, Germany

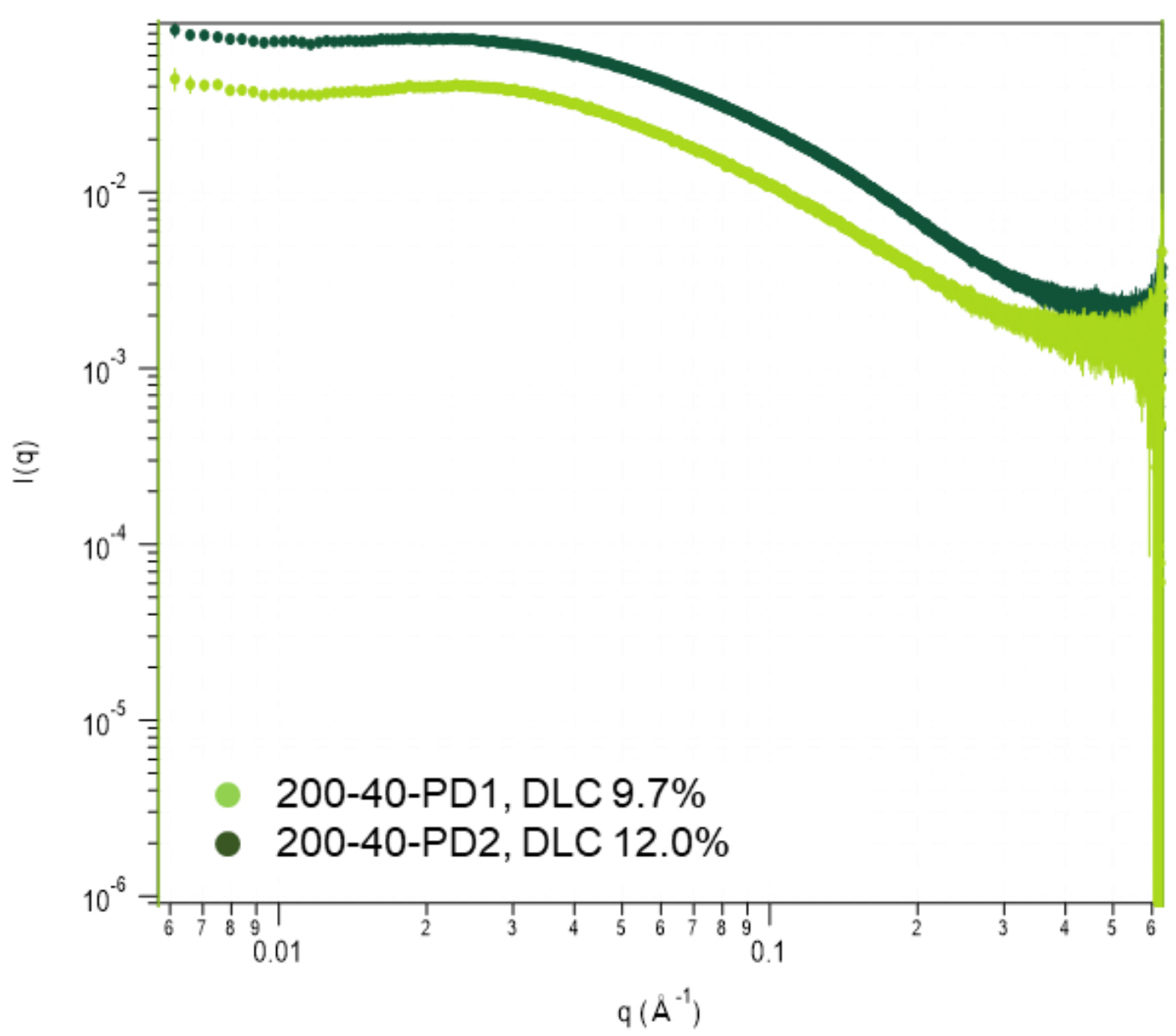

Figure S1. SAXS scattering plot of 200-40-PD1 and 200-40-PD2 in the double logarithmic plot as an example for SAXS scattering data quality, which further was plotted in Guinier plot to determine R_g.

a)

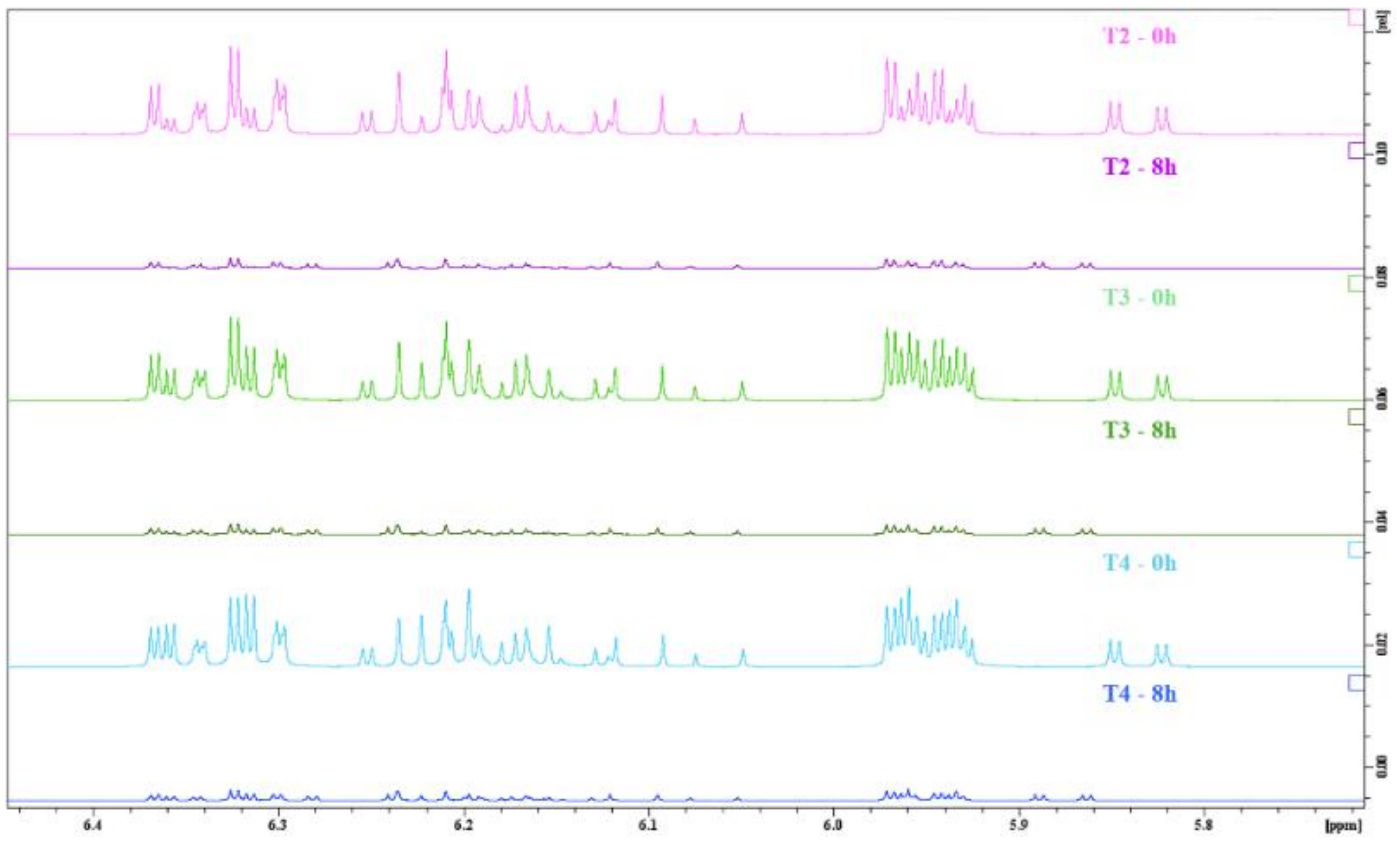
T2 - 0h
T2 - 8h
T3 - 0h
T3 - 8h
T4 - 0h
T4 - 8h
[ppm]
[rel]

b)

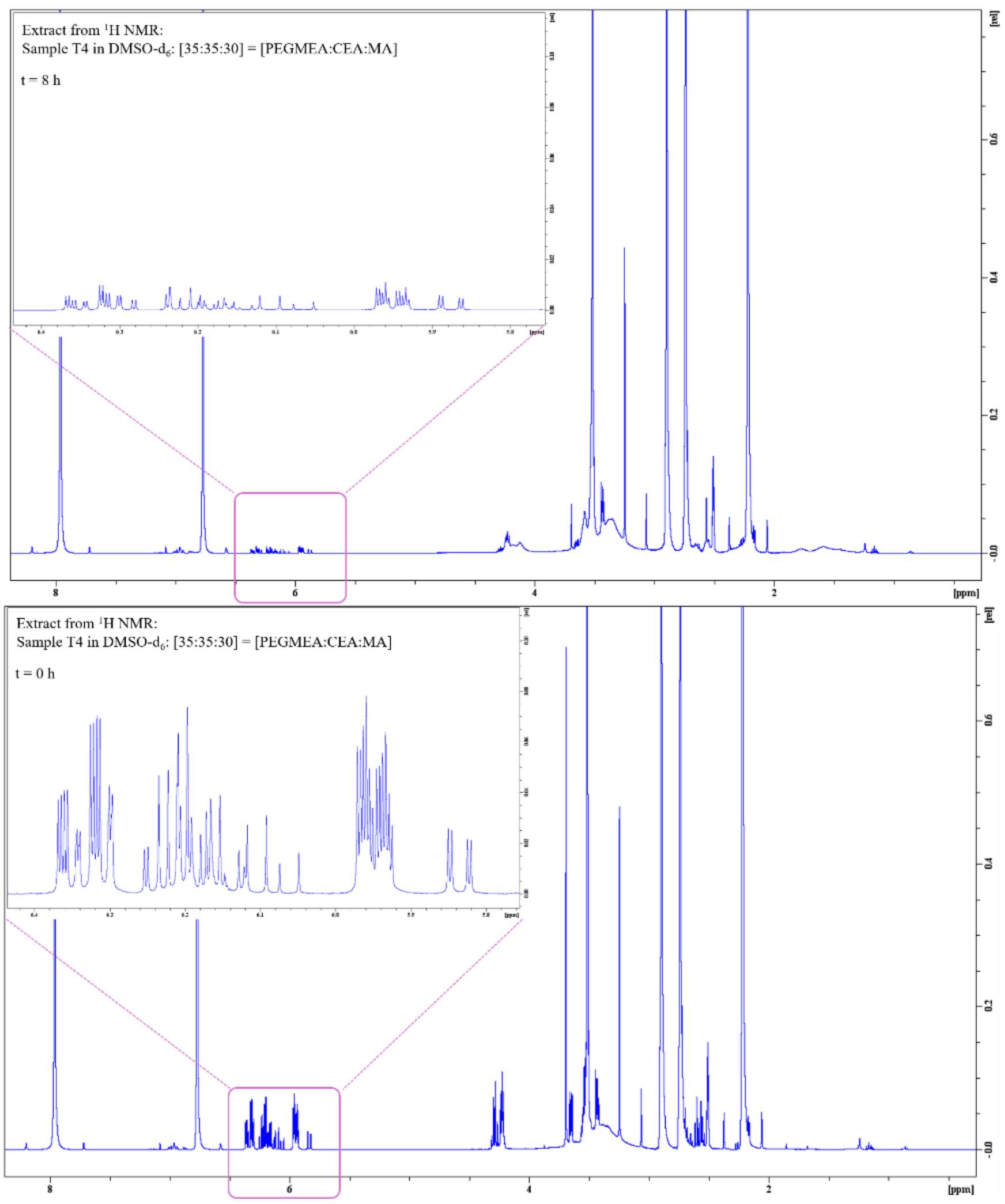
Extract from ¹H NMR:
Sample T4 in DMSO-d6: [35:35:30] = [PEGMEA:CEA:MA]
t = 8 h
Extract from ¹H NMR:
Sample T4 in DMSO-d6: [35:35:30] = [PEGMEA:CEA:MA]
t = 0 h
[ppm]
[rel]

c)

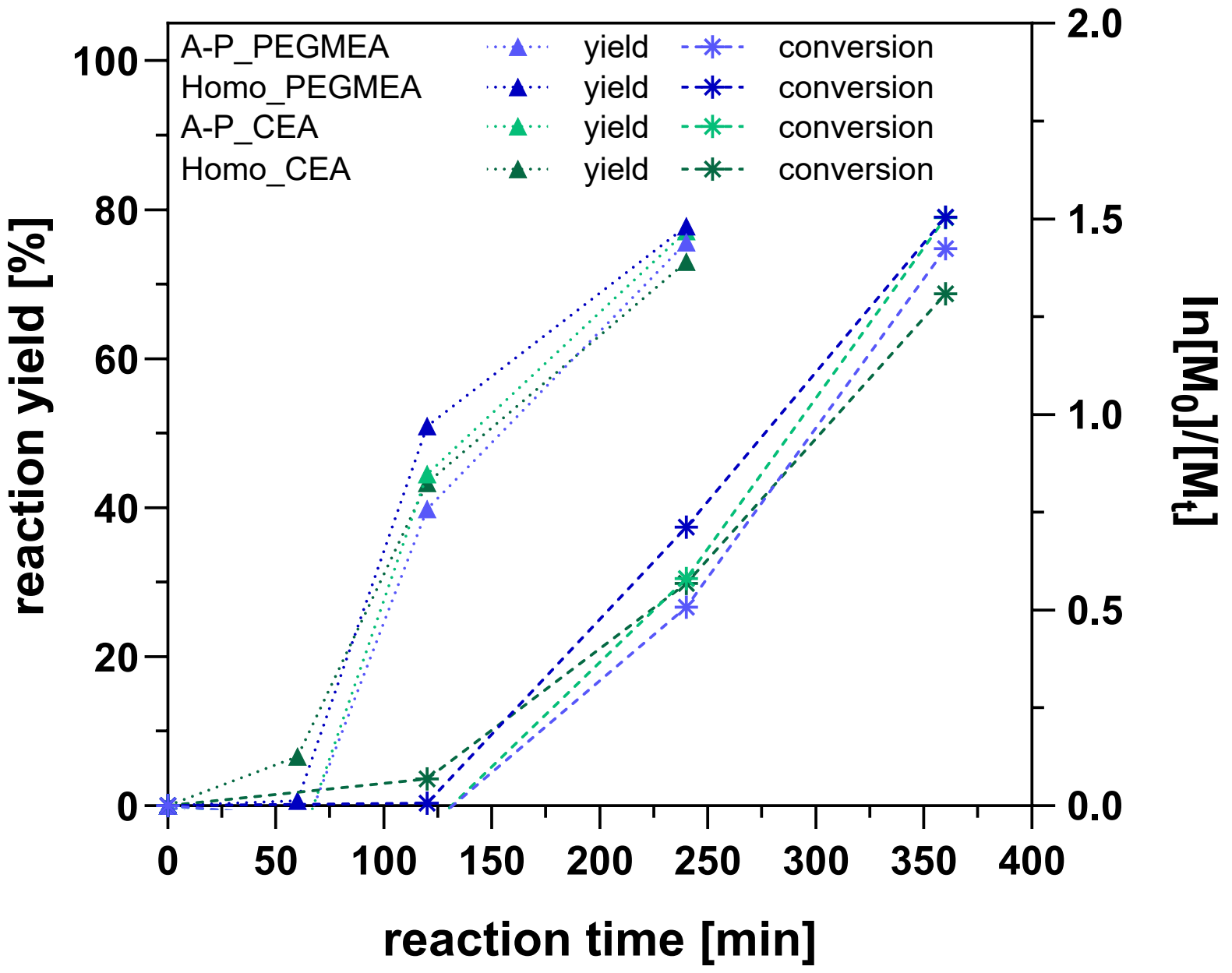

Figure S2. Comparison of the reaction yield and first-order kinetics of PEGMEA and CEA in various copolymerizations (i.e., P(PEGMEA$_{41}$-*co*-CEA$_{42}$)) using NMR analysis (a is magnified while b is the full spectra) to extract each monomer conversion, which was then plotted against time to compare the consumption of the different monomers

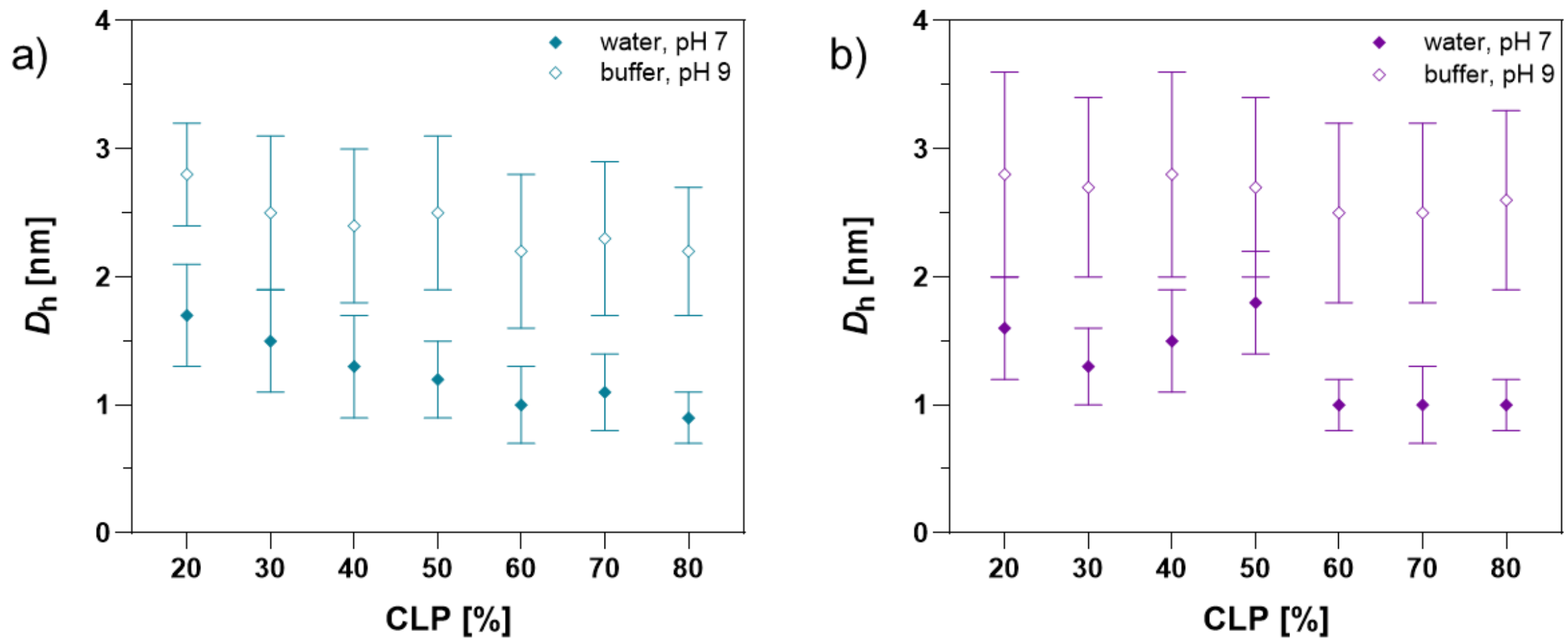

Figure S3. (a, b) Effect of solvent quality on swelling behaviour of P(PEGMEA$_x$-*co*-CEA$_y$) with target DP100 (a) and DP 200 (b) showing larger hydrodynamic diameter of polymer chains when suspended in 0.1M Na_2HPO_4 buffer at pH 8.7-9.3 compared to water at pH 7.

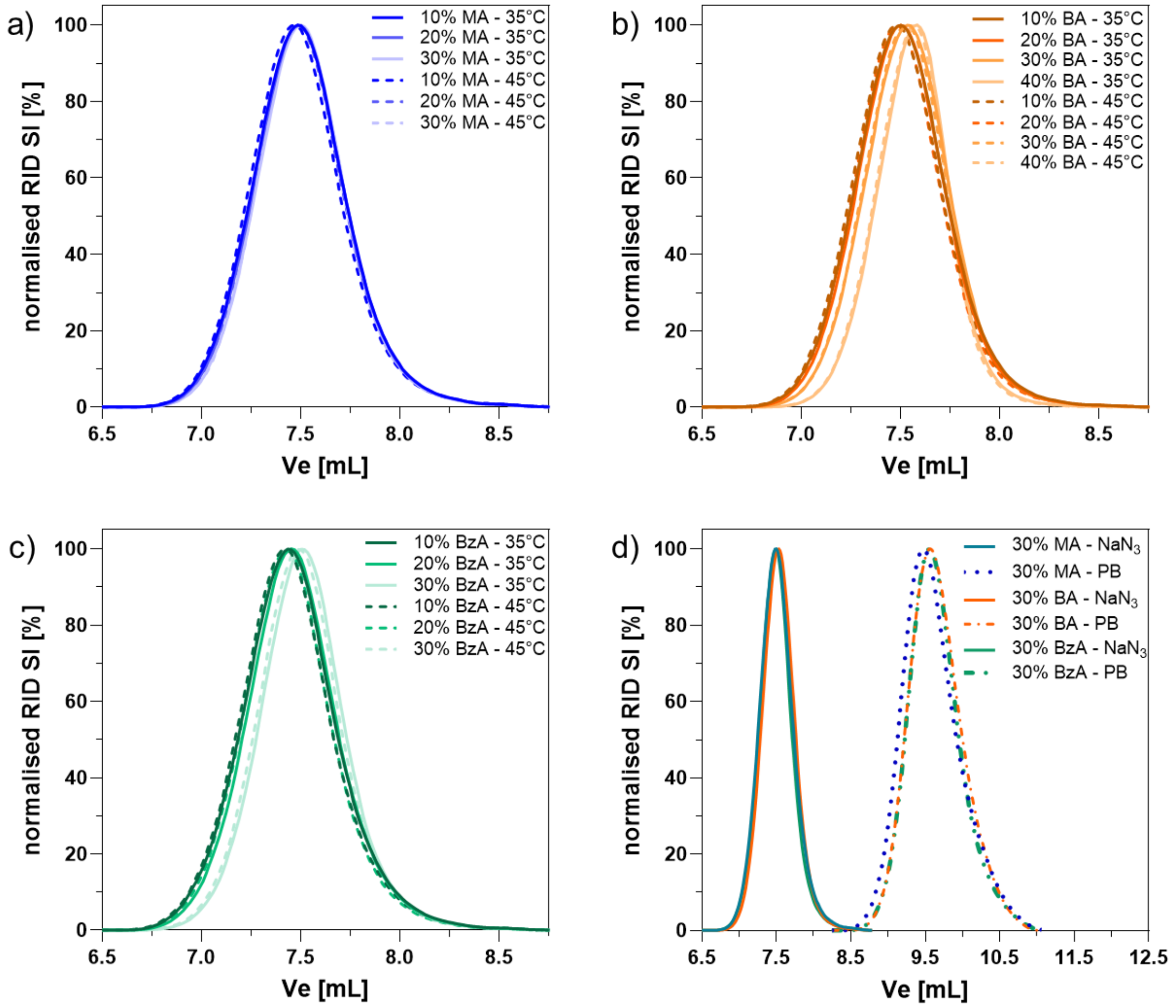

Figure S4. Comparison of SEC-RI chromatograms (SUPREMA linM 5µm column) of polymer samples in 0.2 g$\cdot$L^{-1} NaN_3 varying in the type and the molar percentage of hydrophobic comonomer. Furthermore, the effect of temperature on the elution profile of the samples was tested applying 35 and 45 °C. (a) 10-30 mol% of methyl acrylate (MA). (b) 10-40 mol% of butyl acrylate (BA). (c) 10-30 mol% of benzyl acrylate (BzA). (d) Overlaid chromatograms of 30 mol% of MA, BA, and BzA at 35 °C.

Table S1. Overview of copolymer and polymer-drug conjugates showing repeating units (RU) of PEG, RU of CEA (equals to crosslinking points; CLP), drug to CLP feed ratio, molar drug to CLP ratio after conjugation, drug loading content and efficiency determined via ICP-OES. The number in square brackets is the mol% of CEA, which are the potential crosslinking points CLP

sample name	RU PEG	RU CEA/ [%CLP]	Molar feed ratio (CDDP:COOH)	Molar ratio* (CDDP:COOH)	DLC** [%]	DLE** [%]
100-20-P	71	18				
100-20-PD1		[20]	1:2	1:7.6	1.7	26.5
100-20-PD2			1:1	1:3.6	3.4	26.5
100-30-P	62	27				
100-30-PD1		[30]	1:2	1:2.9	6.8	67.1
100-30-PD2			1:1	1:2.2	8.8	44.6
100-40-P	52	36				
100-40-PD1		[41]	1:2	1:3.5	8.2	57.3
100-40-PD2			1:1	1:2.3	12.2	43.3
100-50-P	41	42				
100-50-PD1		[51]	1:2	1:3.6	10.7	53.8
100-50-PD2			1:1	1:2.4	15.3	41.2
100-60-P	35	54				
100-60-PD1		[61]	1:2	1:2.8	17.3	73.2
100-60-PD2			1:1	1:2.6	18.2	38.1
100-70-P	25	60				
100-70-PD1		[71]	1:2	1:3.1	19.8	63.7
100-70-PD2			1:1	1:3.4	18.6	28.7
100-80-P	16	69				
100-80-PD1		[81]	1:2	1:4.2	19.8	46.4
100-80-PD2			1:1	1:3.7	22.0	26.2
200-20-P	145	38				
200-20-PD1		[21]	1:2	1:5.7	2.3	34.9
200-20-PD2			1:1	1:3.1	4.2	32.9
200-30-P	122	54				
200-30-PD1		[31]	1:2	1:3.2	6.4	63.4
200-30-PD2			1:1	1:2.2	9.0	45.7
200-40-P	103	69				
200-40-PD1		[40]	1:2	1:2.9	9.7	69.8
200-40-PD2			1:1	1:2.3	12.0	44.5
200-50-P	85	88				
200-50-PD1		[51]	1:2	1:3.0	12.8	67.7
200-50-PD2			1:1	1:2.7	13.7	37.0
200-60-P	69	106				
200-60-PD1		[61]	1:2	1:3.5	14.1	56.7
200-60-PD2			1:1	1:2.7	17.8	37.3
200-70-P	52	123				
200-70-PD1		[70]	1:2	1:3.8	16.7	52.5
200-70-PD2			1:1	1:3.0	20.2	32.4
200-80-P	34	141				
200-80-PD1		[81]	1:2	1:4.0	20.2	49.8
200-80-PD2			1:1	1:3.8	21.4	27.2

[*] Molar ratio between cisplatin and carboxylic acid groups on CEA after dialysis. [**] MDL-method detection limit at 0.05 ppm; this is the lowest concentration at which the analyte can be detected in a sample with 99% certainty.

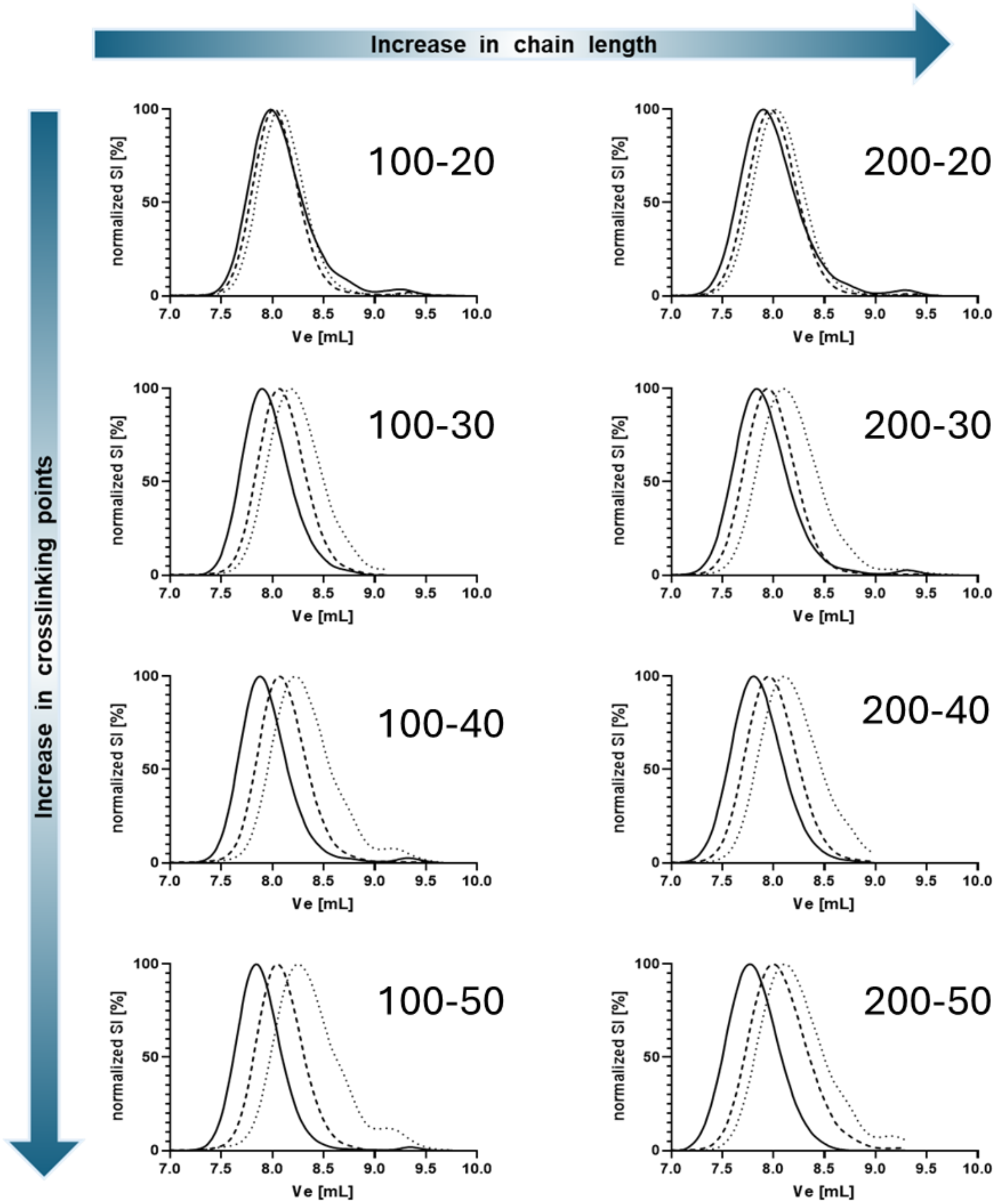

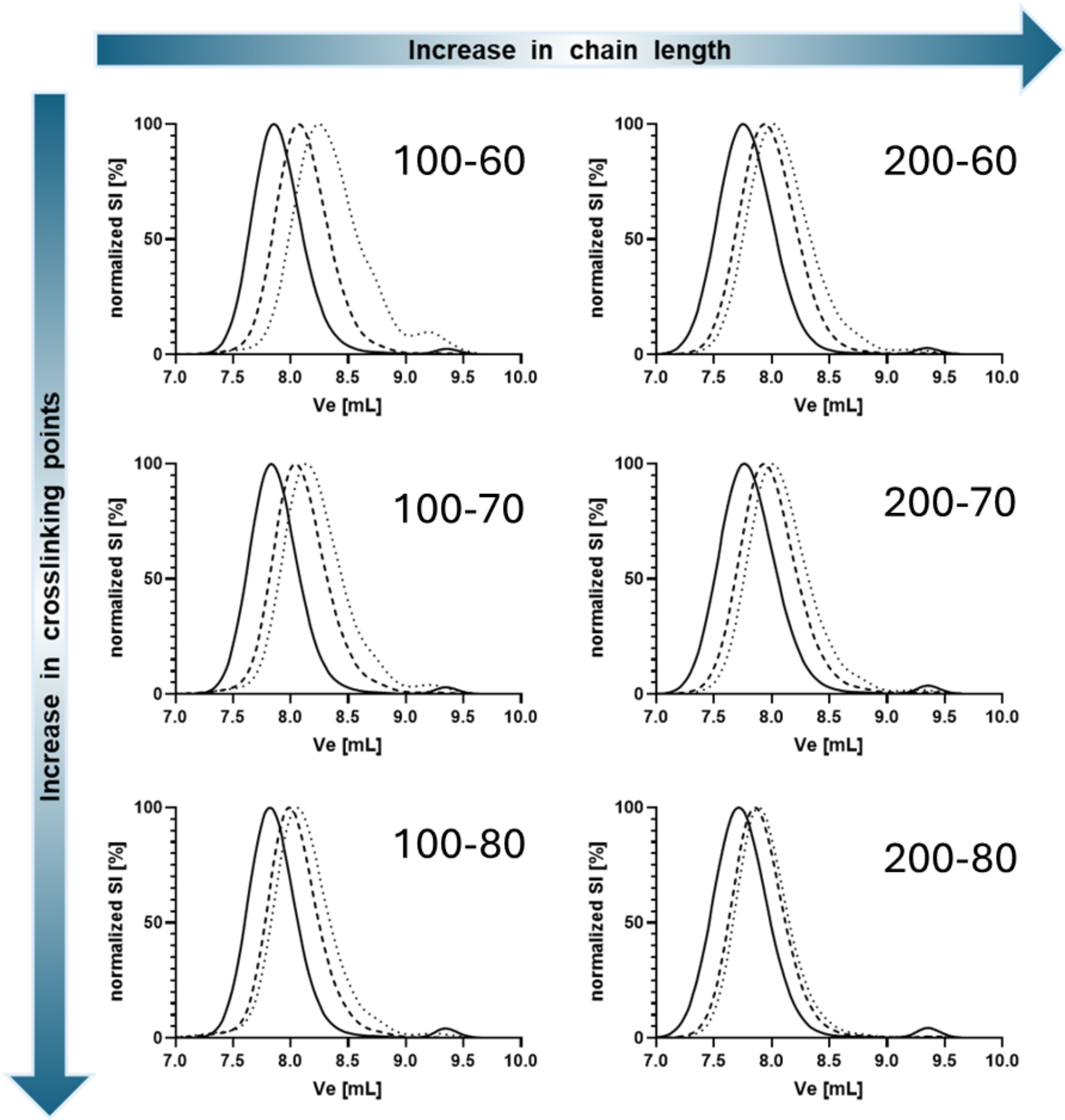

Figure S5. Relative compaction via SEC-RI provides information on whether smaller particles are obtained upon crosslinking P(PEGMEA-*co*-CEA) precursor with cisplatin. Overlays of chromatograms show shifts in RI signals to larger elution volumes comparing polymer precursor with conjugated samples PD1 and PD2 of target DP 100 (left) and comparable DP 200 series (right). Dashed line: PD1 series; Dotted line: PD2 series

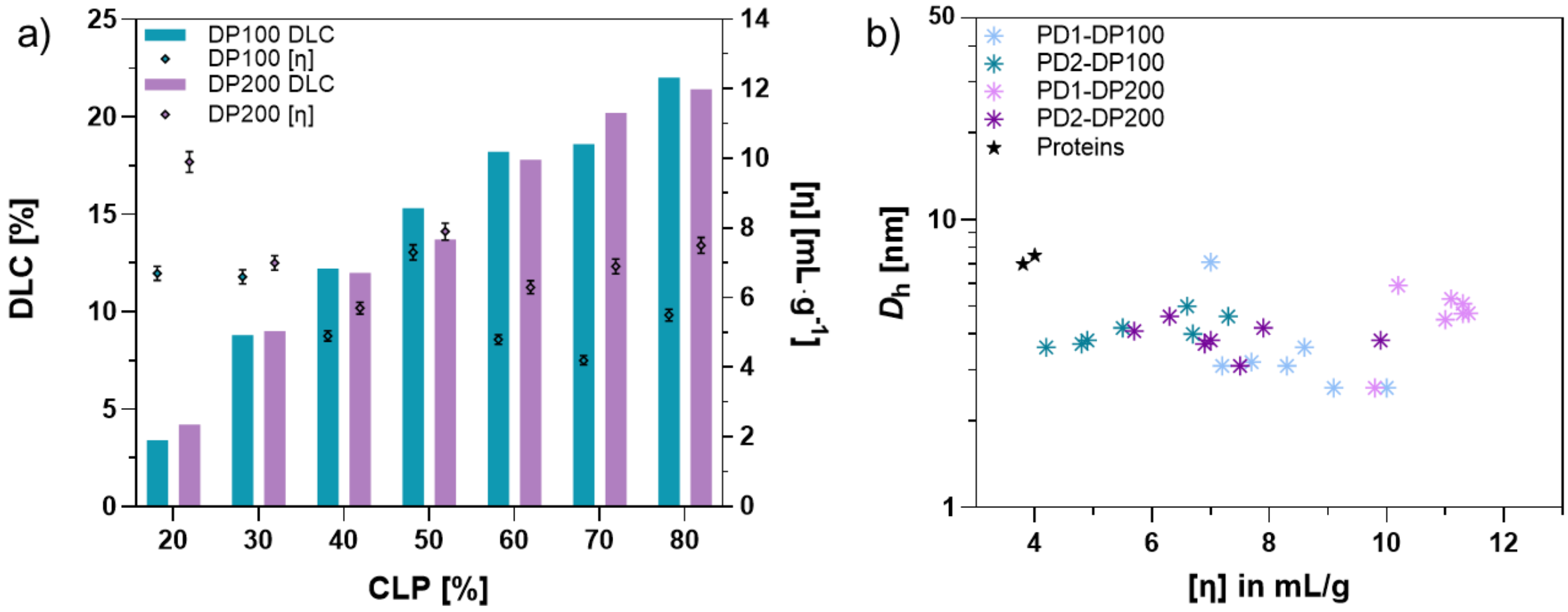

Figure S6. (a) Correlation of drug loading content of cisplatin and the intrinsic viscosity of the PD2 series for DP100 (aqua) and DP200 (purple) to the crosslinking points (CLPs) available on the polymer chains. (b) Compaction as a correlation between dimension and density of PD1 and PD2 series for both DPs and proteins (i.e., BSA[55,56], HSA[56,57]). (c, d) Comparison of R_v of polymer and polymer-drug conjugates (PD1, PD2) for target DP 100 (c) and DP 200 (d).

Table S2. Comparison of DLS-derived R_h in water with 0.02 wt% NaN_3 and 0.1 M phosphate buffer (pH 8.7-9.3) and R_v from Einstein-Simha approximation using viscosity data (in water with 0.02 wt% NaN_3) for P(PEGMEA-*co*-CEA) of target DP 100 and DP 200.

CLP [%]	DP	$R_{h,water}$ [nm]	$R_{h,buffer}$ [nm]	R_v [nm]
**20**	**100**	1.7	2.8	3.7
	**200**	1.8	2.8	4.0
**30**	**100**	1.5	2.5	3.8
	**200**	1.6	2.7	4.1
**40**	**100**	1.3	2.4	3.9
	**200**	1.5	2.8	4.2
**50**	**100**	1.2	2.5	4.1
	**200**	1.3	2.7	4.3
**60**	**100**	1.0	2.2	4.1
	**200**	1.0	2.5	4.4
**70**	**100**	1.1	2.3	4.1
	**200**	1.0	2.5	4.4
**80**	**100**	0.9	2.2	4.1
	**200**	1.0	2.6	4.7

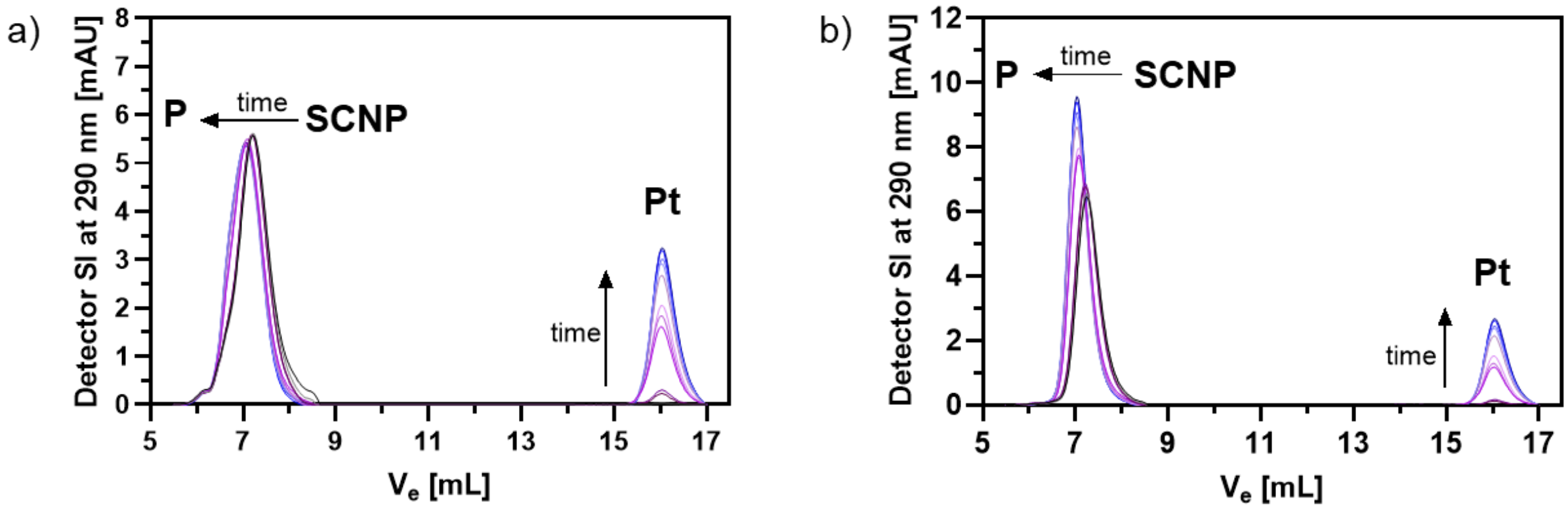

Figure S7. Drug release studies of (a) 200-60-PD2 and (b) T12-PD2 SCNPs over 0 to 108 hours monitored *via* SEC-UV/Vis at 290 nm.

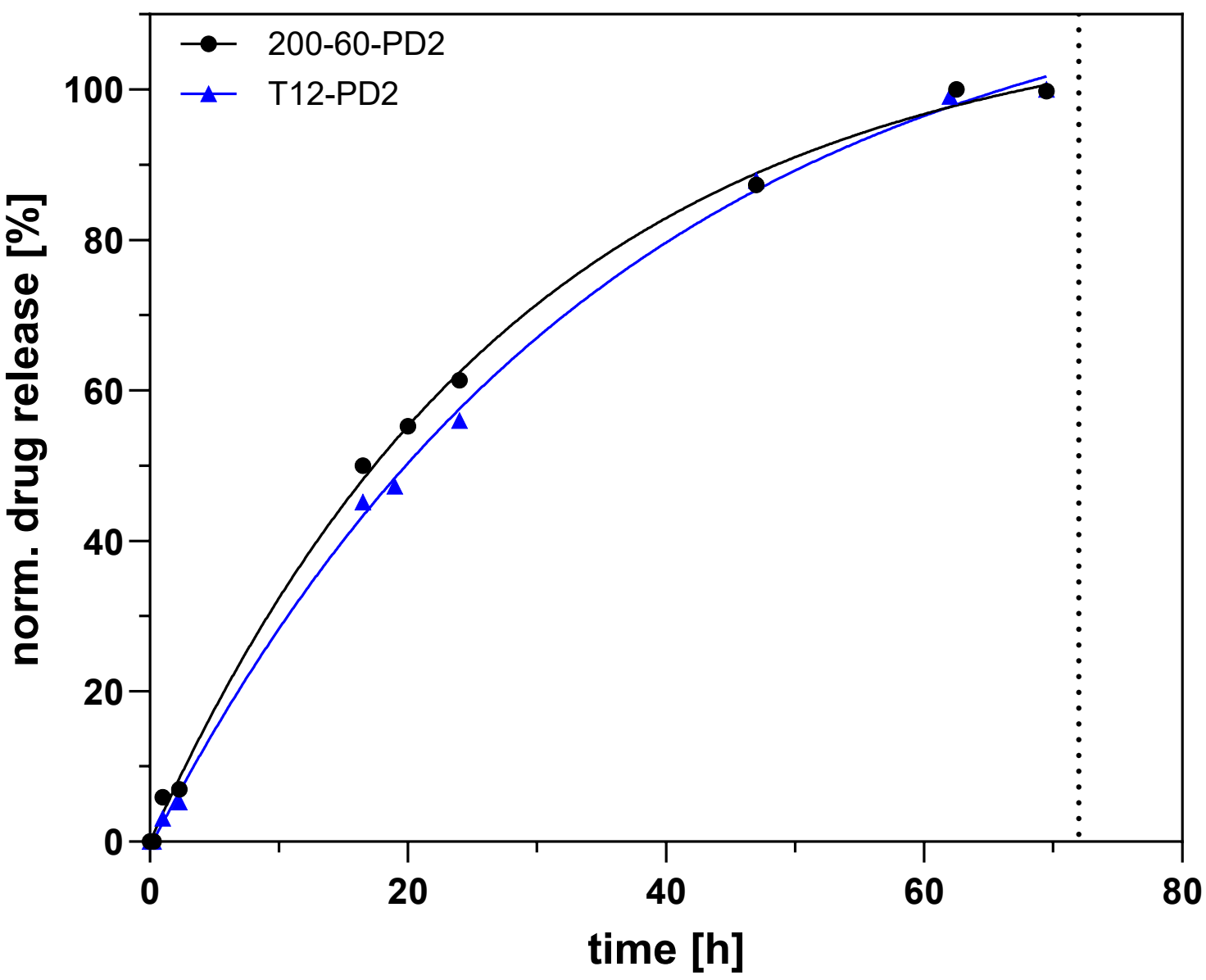

Figure S8. Normalised drug releasing rate of cisplatin from the SCNPs of 60% CLP (200-60-PD2, P(PEGMEA$_{69}$-*co*-CEA$_{106}$) and T12 (T12-PD2, P(PEGMEA$_{63}$-*co*-CEA$_{64}$-*co*-BzA$_{58}$)) over 72 h exposure to 1X PBS at 37 °C.

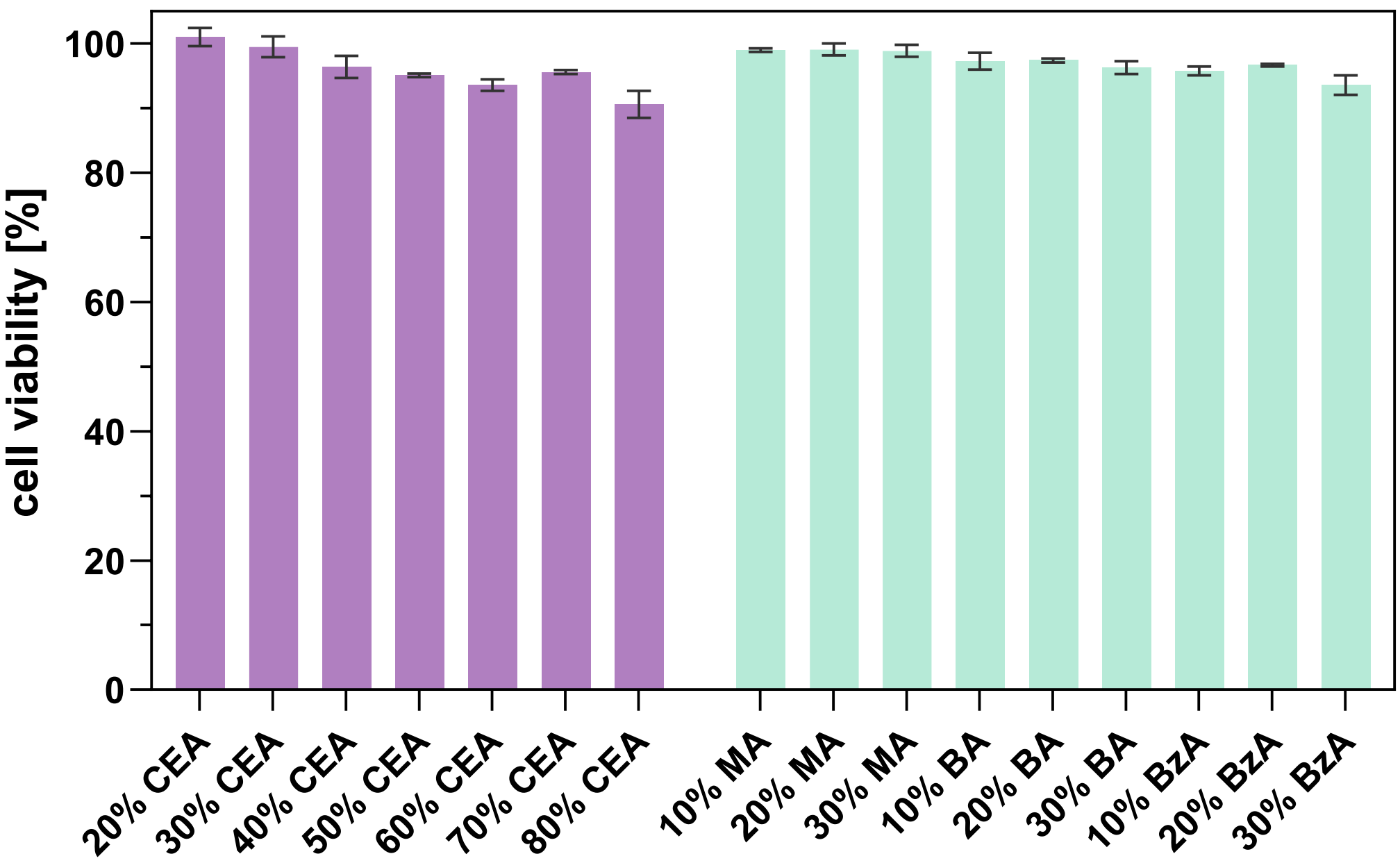

Figure S9. Cell viability of A2780 cells after 72h of exposure to polymer precursors P(PEGMEA-*co*-CEA) and P(PEGMEA-*co*-CEA-*co*-MA/BA/BzA) at 1 mg·mL^{-1}.

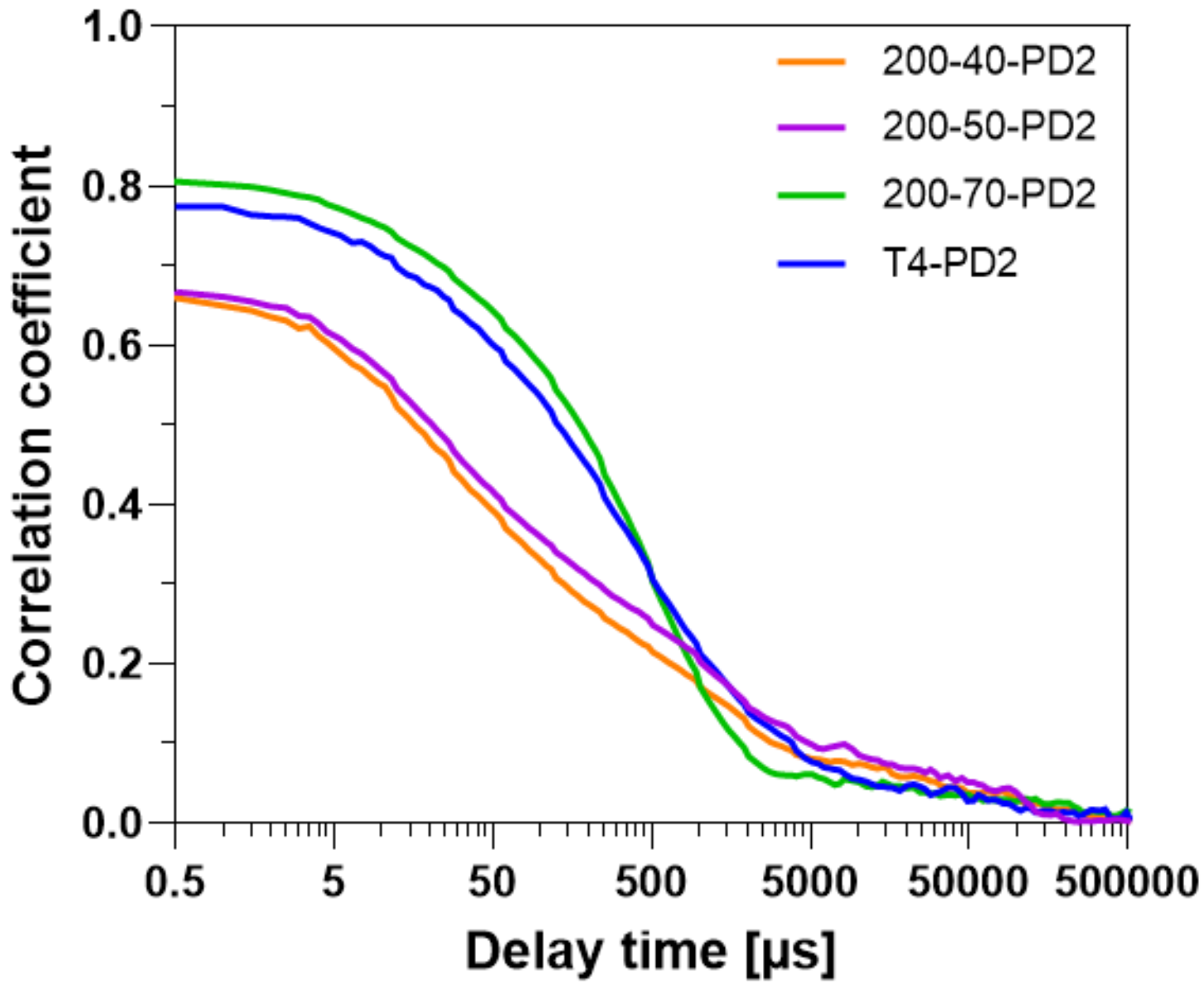

Figure S10. Correlograms of SCNPs 200-40-, 200-50-, 200-70- and T4-PD2 as example for the correlation functions obtained from DLS batch experiments of the herein designed SCNPs.
